# New Material of the Pterosaur *Gladocephaloideus* Lü et al., 2012 from the Early Cretaceous of Liaoning Province, China, with Comments on Its Systematic Position

**DOI:** 10.1371/journal.pone.0154888

**Published:** 2016-06-01

**Authors:** Junchang Lü, Martin Kundrát, Caizhi Shen

**Affiliations:** 1 Institute of Geology, Chinese Academy of Geological Sciences, Beijing, China; 2 Key Laboratory of Stratigraphy and Paleontology, Ministry of Land and Resources, Beijing, China; 3 Department of Ecology, Faculty of Natural Sciences, Comenius University, Bratislava, Slovak Republic; 4 Center for Interdisciplinary Biosciences, Faculty of Science, University of P. J. Safarik, Košice, Slovak Republic; Perot Museum of Nature and Science, UNITED STATES

## Abstract

Although there are nine genera of ctenochasmatoids reported from the Jehol Biota, at present each is known from a specimen that has either a skull or a relatively complete postcranial skeleton. A nearly complete juvenile specimen of *Gladocephaloideus* from the Lower Cretaceous Jiufotang Formation of Sihedang, Lingyuan of Liaoning Province is the most complete ctenochasmatoid preserved to date with a skull and postcranial skeleton. Based on the holotype (IG-CAGS 08–07) and the nearly complete new specimen (JPM 2014–004), the diagnosis of *Gladocephaloideus* is amended: approximately 50 teeth in total with sharp tips; small nasoantorbital opening, occupying approximately 13% of total skull length; ratio of prenarial rostrum length to skull length approximately 0.63; deep groove along the mid-line of the mandibular symphysis; length to width ratio of the longest cervical vertebra = 4.1; ratio of femur length to tibia length = 0.61; tibia as long as the wing-phalange 1. Phylogenetic analysis recovers *Gladocephaloideus* within the clade Ctenochasmatidae. *Gladocephaloideus* has a closer relationship to the Chinese *Pterofiltrus* rather than to other ctenochasmatid pterosaurs. Microstructure of limb bones implies that JPM 2014–004 represents an early juvenile of *Gladocephaloideus jingangshanensis*, and that the type specimen is not a fully grown specimen either. We assume that the holotype may equate to the late juvenile or sub-adult developmental stage of *Gladocephaloideus*.

## Introduction

**Fig 1:**
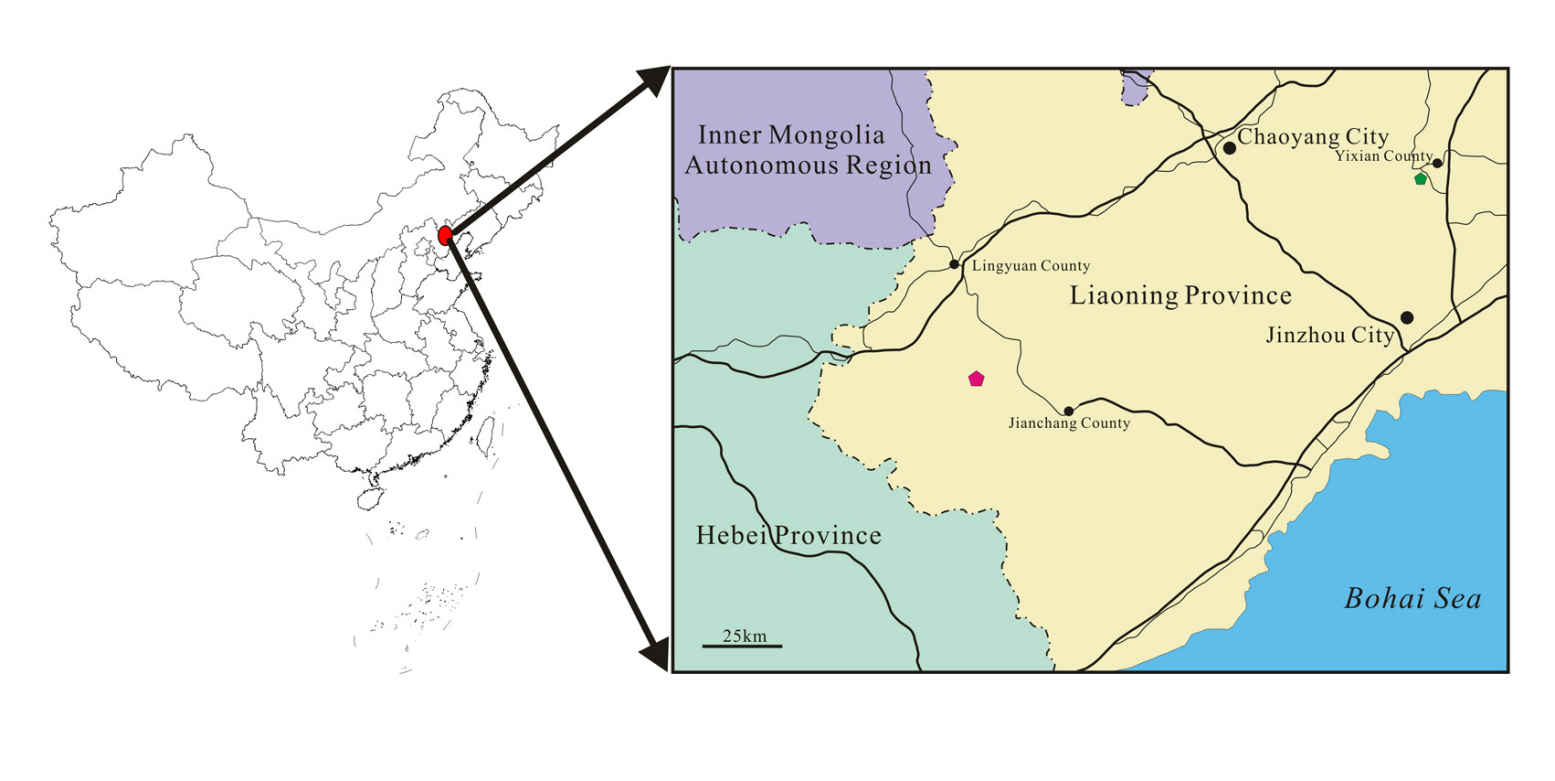
A map of the fossil locality of *Gladocephaloideus jingangshanensis* (JPM 2014–004). The green solid pentagon (near Yixian County) represents the holotype locality.

Ctenochasmatoidea is a group of pterosaurs within the suborder Pterodactyloidea and has been defined as the clade containing *Cycnorhamphus suevicus*, *Pterodaustro guinazui*, their most recent common ancestor, and all of its descendants [1]. At present, although there are nine genera of ctenochasmatoids reported from the Jehol Biota: *Eosipterus* [2], *Beipiaopterus* [3], *Feilongus* [4], *Cathayopterus* [5], *Gegepterus* [6–7], *Elanodactylus* [8–9], *Pterofiltrus* [10], *Gladocephaloideus* [11] and *Moganopterus* [12], they are known from either skulls or a relatively complete postcranial skeletons but not both, making comparisons difficult. A new specimen of a nearly complete juvenile assigned to *Gladocephaloideus* from the Lower Cretaceous Jiufotang Formation of Sihedang, Lingyuan of Liaoning Province (Fig 1) is therefore a significant addition as the most complete ctenocahsmatid yet recovered from this formation. Importantly, it provides new details about skeletal anatomy and development of the genus.

*Gladocephaloideus* was initially assigned to the family Gallodactylidae [11], based on an incomplete skeleton with skull and lower jaw preserved. However, based on the new specimen, the phylogenetic analysis places *Gladocephaloideus* within the clade Ctenochasmatidae, and suggests that it does not belong to Gallodactylidae. The strict consensus tree shows that *Gladocephaloideus* has a close relationship to *Pterofiltrus* rather than to other ctenochasmatid pterosaurs as previously thought [11]. The bone microstructure of the holotype *Gladocephaloideus* indicates that it is not a fully grown individual. However, cortical bone samples suggest initial onset of skeletal maturation. The new specimen (JPM 2014–004) represents much earlier juvenile stage of *Gladocephaloideus jingangshanensis*.

## Materials and Methods

This study was based on the specimen JPM 2014–004, housed in the Jinzhou Paleontological Museum, Jinzhou City, Liaoning Province of China. The senior author (J. Lü) obtained permission from the museum to access the collections. No permits are required for the described study, which is based entirely on museum specimens, and the museum collections are accessible to the public; this research complies with all relevant regulations.

In order to determine the systematic position of *Gladocephaloideus*, a phylogenetic analysis was conducted based on the data matrix of Lü et al.[13] with the addition of five additional taxa: *Gladocephaloideus*, *Guidraco* [14], *Beipiaopterus* [3], *Elanodactylus* [8], and *Pterofiltrus* [10] (S1 File). The character codings for Gl*adocephaloideus* are based on both the holotype *Gladocephaloideus* [11] and the new specimen (JPM 2014–004). The revised data matrix consists of 67 ingroup taxa and 118 characters.

We subjected the dataset to a maximum parsimony analysis in TNT v1.1 [15]. We first conducted a ‘new technology’ search, which recovered a minimum length tree in 10 replicates. This procedure aims to broadly sample tree space and identify individual tree islands. We then subjected the recovered most parsimonious trees (MPTs) to a traditional search with TBR branch swapping, which more fully explores the tree islands found in the ‘new technology’ search. This process returned a total of 3024 MPTs of 461 steps (consistency index = 0.356, retention index = 0.760). Bremer values were used to assess clade support.

In order to investigate bone microstructures, the Gladocephaloideus samples were imbedded in bicomponent epoxy resin (Lamit 109; Kittfort) and ground on a Montasupal grinder (Germany) using silicon carbide (grain size: 400–600 nm). The samples were then re-impregnated with EpoFix (Struers) and fixed before being cut with a diamond blade (150 mm diameter, Struers) and abraded (grits: 240, 400, 600) to 0.2 mm using 1000 grit silicon carbide to reach 30 microns thickness. Finished slides were examined using a transmitted light microscope Leitz DM RXE equipped with operating software Leica LAS V4.2 and a Leica DFC 550 camera, and a polarized light microscopy Leica DM 5500B equipped with operating software LAS AF2.3.5. and a Leica DFC 420C camera. Crossed polarized light reveals the orientation of collagen fiber remains within the bone matrix, thus facilitating its description and interpretation. The images were processed using Adobe Photoshop and CorelDRAW X5 software. Quantitative measurements were made using the measurement software ImageJ.

## Results

### Systematic paleontology

Pterosauria Kaup, 1834 [16]

Pterodactyloidea Plieninger, 1901 [17]

Archaeopterodactyloidea Kellner, 2003 [18]

Ctenochasmatoidea Unwin, 1995 [19]

*Gladocephaloideus* Lü, Ji, Wei, Liu, 2012 [11]

*Gladocephaloideus jingangshanensis* Lü, Ji, Wei, Liu, 2012 [11]

#### Specimen

A nearly complete skeleton with a skull and lower jaws (JPM-2014-004). The specimen is housed in the collections of Jinzhou Paleontological Museum, Jinzhou City, Liaoning Province of China.

#### Locality and horizon

Sihedang, Lingyuan of Liaoning Province, Jiufotang Formation [20].

#### Amended diagnosis

A ctenochasmatoid pterosaur distinguished by the following unique combination of characters: rostrum relatively slender, the distal end of the parietal crest large; about 50 teeth in total, and all the teeth with sharp tips; nasoantorbital opening small, reaching approximately 13% of skull length; ratio of prenarial rostrum length to skull length approximately 0.63; deep groove along the mid-line on the dorsal surface of the mandibular symphysis; length to width ratio of the longest cervical vertebra = 4.1; ratio of femur length to tibia length = 0.61; tibia as long as wing-phalange 1; length ratio of metatarsal III to tibia about 0.4.

#### Description

**Fig 2:**
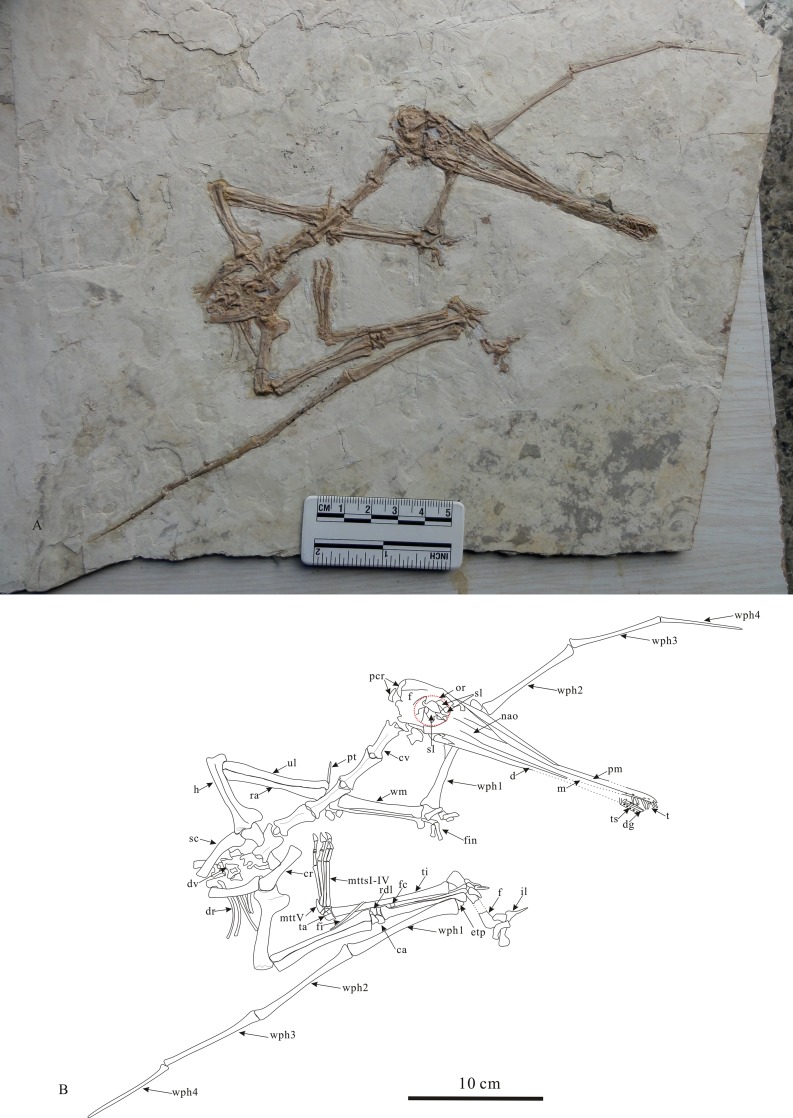
**Photograph (A) and line drawings (B) of *Gladocephaloideus jingangshanensis* (JPM 2014–004).** Abbreviations: ca, carpals; cr, coracoids; cv, cervical vertebrae; d, dentray; dg, deep groove along the mid-line of the mandibular symphysis; dv, dorsal vertebrae; dr, dorsal ribs; etp, extensor tendon process; f, frontal; fc, fifth carpal; fe, femur; fi, fibula; fin, fingers; h, humerus; il, ilium; m, maxilla; mmttsI-IV, metatrals I-IV; mttv, metatarsal V; nao, nasoantorbital opening; or, orbital; pcr, parietal crest; pm, premaxilla; pt, pteroid; ra, radius; rdl, radiale; sc, scapula; st, sternum; sl, sclerotic rings; t, teeth; tc, tooth sockets; ti, tibia; ul, ulna; wm, wing metacarpal; wph1-4, wing phalanges 1–4. Scale bar = 5 cm.

**Fig 3:**
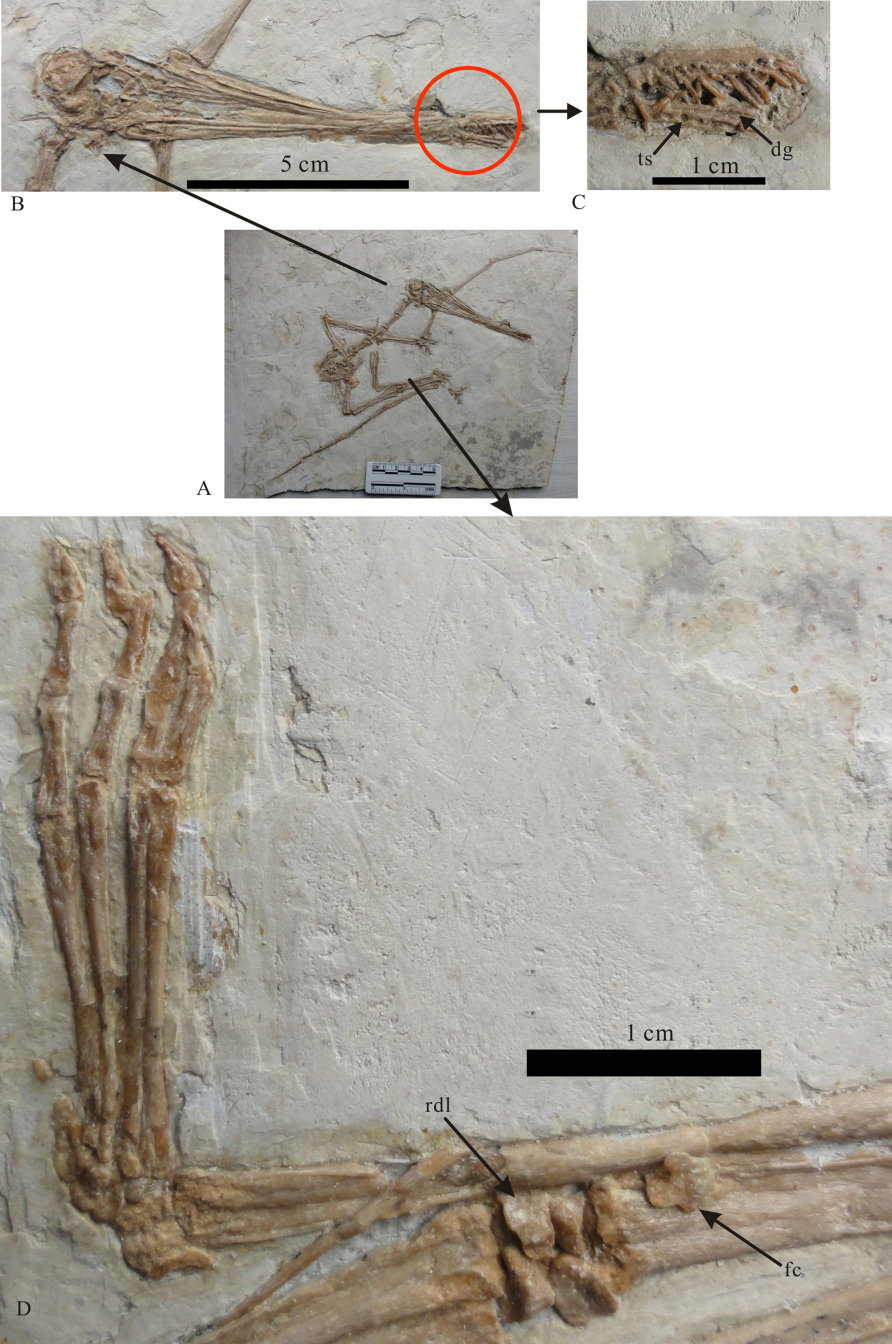
*Gladocephaloideus jingangshanensis* (JPM 2014–004). (A) Overview of the skeleton; (B) Close up of the skull; (C) Close up the anterior part of the rostrum, showing the deep groove along the mid-line of the mandibular symphysis; (D) Close up of the left pes and the right carpals. Abbreviations: dg, deep groove along the mid-line of the mandibular symphysis; fc, fifth carpal; rdl, radiale; ts, tooth sockets.

The skeleton is almost completely preserved except for lacking the posterior dorsal vertebrae, sacral vertebrae and the left hindlimb (Figs 2 and 3; see S1 File). The skull is complete. The orbit is round with a diameter of 10.9 mm and bears a distinct ridge along the dorsal and posterior margin (Fig 3B), which is located above the level of dorsal margin of the antorbital fenenstra. The antorbital opening is small (Fig 2), which is similar to that of the holotype. The teeth are slender and restricted to the anterior end of the rostrum. The teeth of the upper jaw are directed anteroventrally. The posterior portion of the lower tooth rows is broken, thus the exact number of the teeth is not clear. But according to the holotype [11], and the distance relationship between the teeth, the tooth number is inferred to be 50 teeth in total (26 upper jaw teeth and 24 lower jaw teeth). The parietal crest is round and distinct, similar to that of the holotype specimen of *Gladocephaloideus jingangshanensis* [11]. The anterior portion of the lower jaw broken, but it was glued back wrong direction. The approximate width of the mandibular symphysis is 2.8 mm. However, a deep groove on the midline of the dorsal surface of the mandibular symphysis is clear (Fig 3C).

Vertebrae: There are six cervical vertebrae visible (#3-#8). The atlas-axis complex is most likely buried beneath the skull. The mid-series of cervical vertebrae are relatively elongate. The length -to- width ratio of the longest cervical vertebra (the third cervical vertebra) is about 4.14. The neural spines of the cervical vertebrae are considerably low, weak and ridge-like. The seventh cervical vertebra is shorter than those anterior to it. The eighth cervical vertebra, which exhibits a transitional morphology, is relatively broad and low anteriorly, but narrow and tall posteriorly, as is typical at this position in the vertebral column of pterosaurs (Bennett, 2014), and it overlies the anterior edge of the sternum. Cervical vertebrae do not appear to exhibit pleurocoels. Cervical ribs are absent. There are three incomplete dorsal vertebrae preserved. The anterior-most dorsal, equivalent to position 9 in the vertebral column, seems to be lost. Five dorsal ribs are preserved on the right side. The first two ribs are shorter and stouter than the following three. The fourth and fifth dorsal ribs are relatively slender and thread-like.

Pectoral girdle: The scapula is longer than the coracoid. These bones are not fused, indicating osteological immaturity. The proximal termination of the scapula is round and slightly expanded. The shaft of the scapula is curved with a deep, concave medial profile and slightly convex lateral profile. As is typical for pterosaurs, the distal end of the scapular shaft located adjacent to the 13^th^ vertebra on the slab, is not expanded. The shaft of the coracoid is straight, and the proximal end is expanded. The distal end of the coracoid bears a distinct articular surface for the sternum.

Sternum: The sternum is exposed in dorsal view and partially covered by some dorsal vertebrae. The sternum is fan-shaped and bears a distinct cristospine.

Humerus: The humerus is 30.0 mm long with a 4.0 mm width of its shaft. The shaft of the humerus is slightly curved in lateroposterior view. The semicircular deltopectoral crest is longer proximodistally (parallel to the shaft) than lateromedially (it is vertical to the shaft of the humerus). There is a weak ridge near the lateral margin of the deltopectoral crest. The posterior margin of the humeral head is directed posteriorly. The distal end of the humerus bears two condyles, the lateral is larger than the medial. The distal end surface bears two articular facets that contact the ulna and radius respectively. There is no pneumatic opening on the posterior surface of the humeral head.

Ulna and radius: Both right and left ulna/radius are well-preserved. The radius is slightly longer than ulna. The ulna and radius are 39.1 mm and 40.0 mm long respectively. The width ratio of the shafts of the ulna to radius is 1.33. The ulna and radius are both straight elements and lie parallel to each other. The proximal ends of ulna and radius are not well-ossified, but both ends of the ulna are clearly expanded. There is a weak condyle on the lateral surface of the proximal end of the ulna. Both ends of the radius are slightly expanded.

Pteroid: The right pteroid is well-preserved. It is 16.8 mm long. The length ratio of the pteroid to humerus is 0.56. Its proximal portion is slightly curved medially. The distal end of the pteroid is pointed.

Carpals: There are five carpals (Fig 3D). Four are arranged in two rows that are preserved between the distal ends of the ulna/radius and the proximal ends of metacarpals. The proximal row is composed of two carpals. They are irregular in shape and contact the proximal end of the radius. The lateral element is triangular in dorsal view and covers parts of the proximal ends of radius and ulna. The medial element (radiale) is irregularly quadrilateral in dorsal view. Its dorsal surface is concave with raised margins, and the lateral surface of the medial carpal bears a distinct shallow groove that articulated with the lateral one. Its proximal side also bears a distinct concave surface for articulation with the proximal end of the radius. The distal surface of the medial carpal articulated with the distal carpals.

There are three distal carpals. Two of them form the distal row. The lateral element is the largest of the carpals in dorsal view, and it is an irregular pentagon in shape with a slightly concave dorsal surface. The medial carpal articulates with the radiale. There is a small opening located near the center of the carpal. The distal side of this element articulates with the lateral portion of the proximal end of the wing metacarpal. The fifth carpal is displaced and lies along the surface of the metacarpal IV. It is the smallest of the carpals and is anteroposteriorly shorter than it is lateromedially wide. It is cylindrical in shape with a constricted middle portion. The proximal end of this carpal is concave, with a distinct articular surface.

Metacarpals: The first left metacarpal is slightly displaced from its original position, the second is preserved in its original position and the third is covered by the left wing metacarpal. The second and third metacarpals are relatively long compared with those in more derived pterosaurs in which metacarpals I-III are very short compared to the wing metacarpal. The length ratio of the metacarpal I to the wing metacarpal IV is 0.90. The wing metacarpal is 32.4 mm long, and it is straight with a greatly expanded proximal end and a slightly expanded distal end. The proximal end of the wing metacarpal is flattened, and its distal end bears a distinct trochlea.

The first three fingers of the left manus are not well preserved, and all the phalanges are disarticulated. There is a single manual ungual preserved. It is slightly curved with a sharp distal end.

Wing phalanges: The four wing phalanges become progressively smaller distally. The extensor tendon process is not fused with the proximal end of wing phalange 1. The epiphyses of the wing phalanges are poorly ossified. The dorsal surfaces of the wing phalanges are smooth.

The pelvic girdle is incomplete. Only a portion of the ilium and proximal parts of the pubis and ischium of the left side are preserved. All of these pelvic elements are uninformative due to the poor preservation.

Femur: The right femur is not preserved. The left femur is missing its middle portion but its length can be measured (it is 27.5 mm long). It is curved anteriorly. The distal end of the femur is slightly expanded and relatively poorly ossified. The femoral head is supported by a distinct, stout neck. The angle formed by the neck and the femoral shaft is about 135°. The femur is stout, and reaches about 61% of the tibia length. There is a foramen on the posterior surface near the proximal end of the femur.

Tibia and Tarsals: The right tibia is well-preserved. The tibia is straight and nearly as long as the wing phalange 1. Both the proximal and distal ends are poorly ossified. The tarsals are not well ossified either, and it is difficult to assess their morphology.

Metatarsals and digits (Fig 3D): The first and second metatarsals (it is 17.8 mm long) are equal in length. The third metatarsal (it is 18.6 mm) is the longest of the metatarsals, and the fourth metatarsal is the shortest. The second to fourth toes are nearly equal in length. The pedal phalangeal formula is 2-3-4-5-0. The second and third phalanges of the fourth toe and the second phalange of the third toe are not well ossified. The pedal unguals are relatively small with sharp tips.

## Comparison and Discussion

At present, nine genera of ctenochasmatoid pterosaurs are known from the Yixian Formation. Some, such as *Beipiaopterus*, *Eosipterus* and *Elanodactylu*s, are missing skulls, and it is difficult to make comparisons with the holotype of *Gladocephaloi*deus (IG-CAGS 08–07), which comprises only the skull and partial postcrania [11]. The new specimen which preserves the with skull and a nearly complete postcranial skeleton facilitates comparison with other ctenochasmatoid taxa. Some comparisons with the related ctenochasmatid pterosaurs were made previously [11], and these not be repeated here.

The unfused contact between the extensor tendon process and the proximal end of wing phalange 1, as well as the poorly ossified epiphyses of the wing phalanges, indicates that JPM-2014-004 is an early juvenile individual

*Gladocephaloideus* differs from *Eosipterus* [2] in that the length ratio of ulna to wing metacarpal is 1.23, smaller than that of *Eosipterus* (1.3 [2]). The lengths of the ulna, wing phalange 1 and tibia are nearly equal in *Eosipterus*, whilst in *Gladocephaloideus* the ulna is shorter than wing phalange 1, which is slightly longer than the tibia. The length ratio of femur to tibia is 0.67 in *Eosipterus*, whilst this ratio is 0.61 in *Gladocephaloideus*.

*Gladocephaloideus* differs from *Beipiaopterus* [3] in that *Beipiaopterus* has three wing phalanges; the length ratio of femur to tibia is 0.41, which is much smaller than that of *Gladocephaloideus* (0.61); and wing phalange 1 is much longer than the tibia in *Beipiaopterus* [3], whilst it is slightly longer than the tibia in *Gladocephaloideus*. The ratio of metatarsal III to tibia of *Beipiaopterus* is 0.37, which is smaller than that in *Gladocephaloideus* (0.41).

*Gladocephaloideus* differs from *Elanodactylus* [8–9] in that both the second and third wing phalanx of *Elanodactylus* are longer than the first, and the second wing phalanx is the longest bone in the wing. The lengths from the first to third wing phalanges are reduced, and the wing phalanx 1 is the longest bone in *Gladocephaloideus*.

*Gladocephaloideus* differs from *Feilongus* [4] in that two sagittal cranial crests are present in *Feilongus*, whilst *Gladocephaloideus* does not bear a cranial crest; there are total of 76 teeth in *Feilongus*, which is greater than that in *Gladocephaloideus* (total of 50 teeth) [11].

*Gladocephaloideus* differs from *Monganopterus* [12] in that *Monganopterus* is the largest toothed pterosaur with skull length longer than 95 cm, and it bears a long, narrow, blade-like parietal crest and there are totally about 62 long, slender, curved teeth; whilst *Gladocephaloideus* does not bear a blade-like parietal crest and only bears totally about 50 teeth.

*Gladocephaloideus* differs from *Pterofiltrus* [10] in that there are about 50 teeth in total, and the dentition occupies 24% the skull length. In contrast, *Pterofiltrus* has about 110 teeth in total, and the dentition occupies 50% of the skull length.

## Phylogenetic Analysis

JPM-2014-004 is assigned to *Gladocephaloideus* based on the following characters: the skull bears a slender rostrum, and the distal end of the parietal crest is round; the nasoantorbital opening is small, occupying approximately13% of the skull length; ratio of prenarial length to skull length is approximately 0.63 and the length ratio of metatarsal III to the tibia is about 0.4 [11]. The scores of *Gladocephaloideus* for phylogenetic analysis are based on the holotype (IG-CAGS 08–07) and an early juvenile individual (JPM 2014–004). The present analysis includes all nine genera of ctenochasmatoids reported from the Jehol Biota: *Eosipterus* [2], *Beipiaopterus* [3], *Feilongus* [4], *Cathayopterus* [5], *Gegepterus* [6], *Elanodactylus* [8], *Pterofiltrus* [10], *Gladocephaloideus* [11] and *Moganopterus* [12]. Although most of these generaare not represented by complete specimens, they still provide important information for analysis.

**Fig 4:**
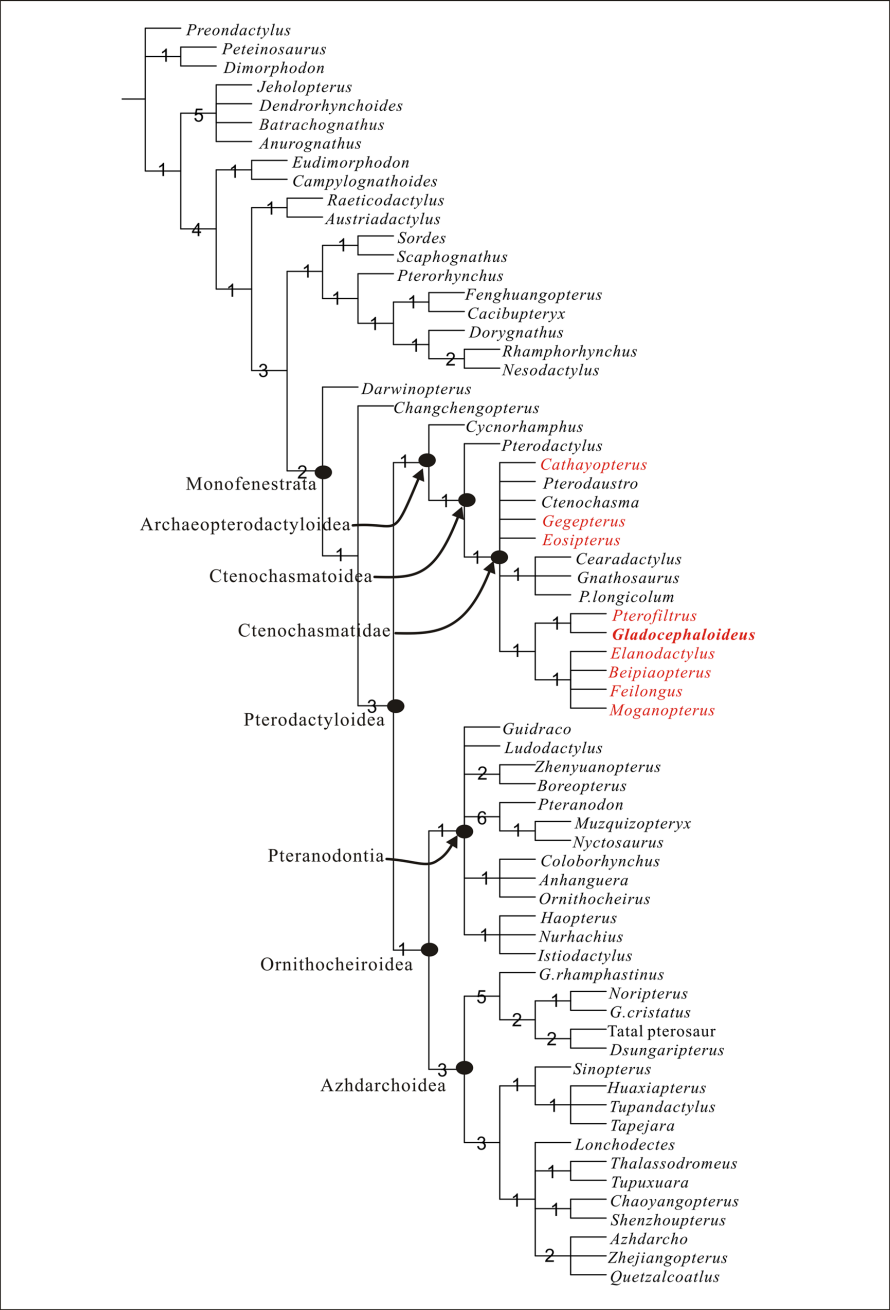
Strict consensus of 3024 most parsimonious trees obtained by TNT, based on analysis of 67 ingroup and 117 characters, showing the phylogenetic position of *Gladocephaloideus jingangshanensis* (Tree length = 461, consistency index = 0.356 and retention index = 0.760). Character and state distributions at key nodes are as follows: Monofenestrata, 6 (2), 13(2), 14(2), 17(1), 20(1), 95(0), 118 (1); Pterodactyloidea, 66 (1), 70(1),71(1),72(0),89(3),116(1); Archaeopterodactyloidea,29(1), 30(1), 32(1), 34(2), 64 (1),104(1); Ornithocheiroidea,63(1), 87(1), 93(1); Pteranodontia,22(1),25(1), 69(1), 78(1), 86 (1), 89 (3),90 (1), 95 (1), 98 (2), 108(1), 113 (2). Ctenochasmatoidea, 65(1), 67(1), 68(2); Ctenochasmatidae,5(1), 60(1); Azhdarchoidea, 47 (0), 54(1), 107(1), 110(1), 112 (1). Numbers adjacent to each node are Bremer support values. Liaoning ctenochasmatid pterosaurs are in red.

A phylogenetic analysis including 67 ingroup taxa and 118 characters recovers 1296 most parsimonious trees. The strict consensus of the 1296 most parsimonious trees (Fig 4) recovers all the Liaoning ctenochasmatid pterosaurs within the clade Ctenochasmatidae and shows that *Gladocephaloideus* is more closely related to *Pterofiltrus* than to other ctenochasmatoids. *Gladocephaloideus* and *Pterofiltrus* form a clade supported by one synapomorphy: the antorbital fenestra is at least as twice as long as it is deep. The apomorphies of *Gladocephaloidus* among ctenochasmatoids are: posterior region of skull is rounded; squamosal position is entirely below the orbit; occiput faces ventrally; quadrate is subhorizontal and lateral pneumatic foramen on centrum of the cervical is absent. The synapomorphies of *Gladocephaloideus* among ctenochasmatid pterosaurs are: the nasal process of maxilla is vertical-subvertical; the frontal extends anterior to the lacrimal-jugal bar; and fronto-parietal crest is flange-like and short. *Elanodactylus*, *Beipiaopterus*, *Feilongus* and *Moganopterus* form a clade, which shares one character: mid-series cervicals are very elongate. Bremer supports from 6562388 trees indicate that each node is well-supported (Fig 4).

## Developmental Changes in Bone Histology of *Gladocephaloideus*

The samples were collected from the holotype of *Gladocephaloideus jingangshanensis* (IG-CAGS-08-07) and the smaller individual which is housed in the collections from the Jinzhou Paleontological Museum (JPM 2014–004). JPM 2014–004 is represented by a single sample extracted from the mid-shaft of the left tibia, and the larger specimen (IG-CAGS-08-07; holotype) is represented by two samples: from a lower part of the diaphysis of the left tibia (counter-slab), and from an upper part of the diaphysis of the right tibia (counter-slab).

We present here a detailed description for each sample collected from JPM 2014–004 and IG-CAGS-08-07. The samples usually display approximately a quarter of the overall cross-sectional plane of the sampled bones. In most cases, we show images of areas devoid of large breaks and micro-cracks. However, both more and less intact areas of the bone sections were equally analyzed. In general, microstructural preservation is not heavily altered by diagenesis, and therefore it allowed us to discern most histological details. The terminology used in our histological description follows that of Francillon-Vieillot et al. [21].

**Fig 5:**
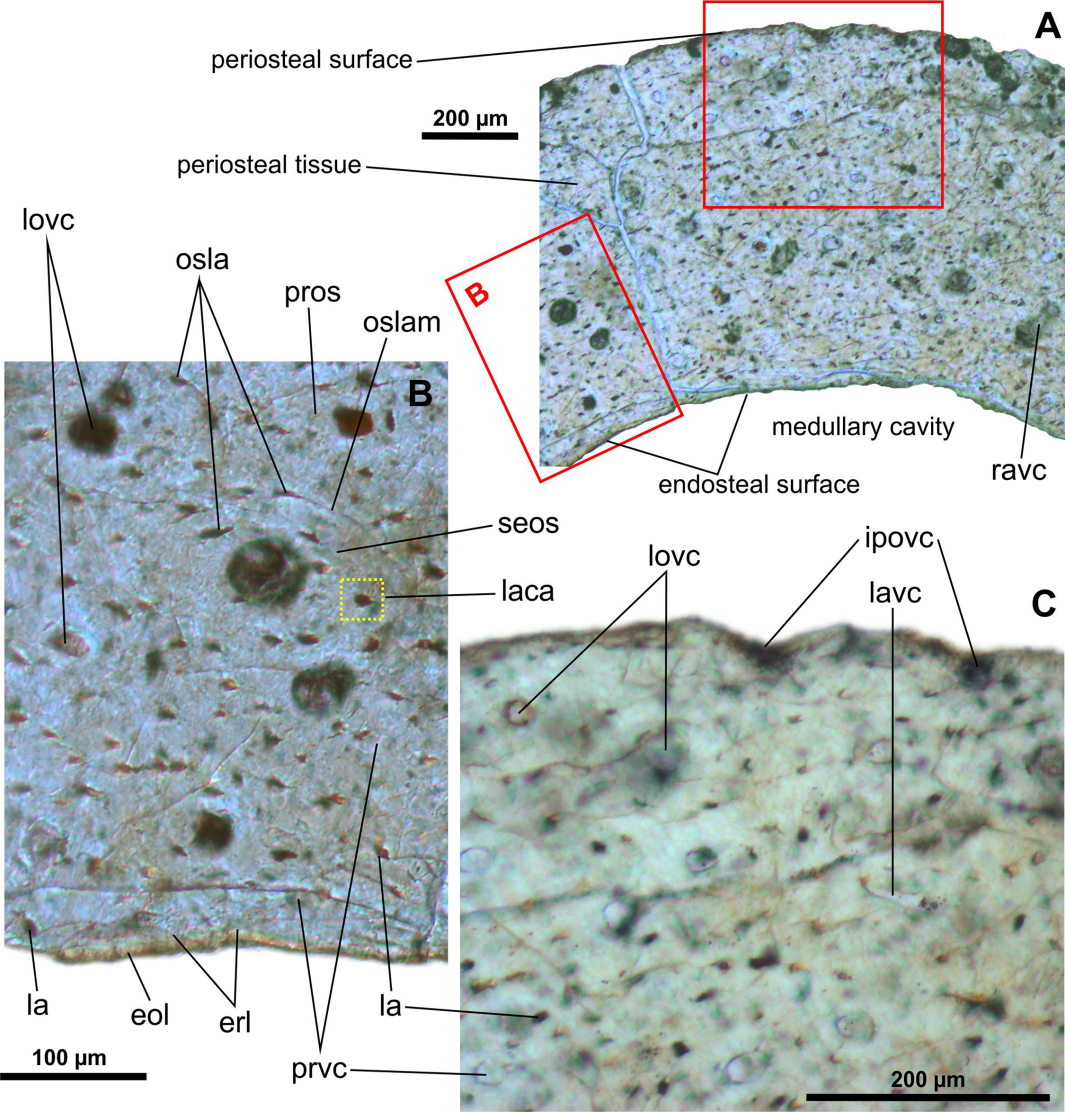
Transverse histological sections of the tibia of an early juvenile individual of *Gladocephaloideus jingangshanensis* (JPM 2014–004) viewed with transmitted light microscopy. **A.** The cortex of the midshaft of the left tibia composed of numerous premature primary osteons enclosing longitudinal vascular canals. **B.** Close-up of the inner midshaft cortex showing presence of a single secondary osteon and bulky osteons with radially projected lacunar canaliculi; note thin endosteal bone that rims the medullary cavity. **C.** Close-up of the outer midshaft cortex formed by osteons randomly embedded in the woven bone matrix; note that some canals are open onto the outer periosteal surface. Abbreviations: eol, endosteal layer; erl, endosteal resorption line; ipovc; incomplete periosteal vascular canal; la, osteocyte lacuna; laca, lacunar canaliculi; lavc, laminar vascular canal; lovc, longitudinal vascular canal; osla, osteonal lacuna; oslam, osteonal lamella; pros, primary osteon; prvc, primary vascular canal; ravc, radial vascular canal; seos, secondary osteon.

**Fig 6:**
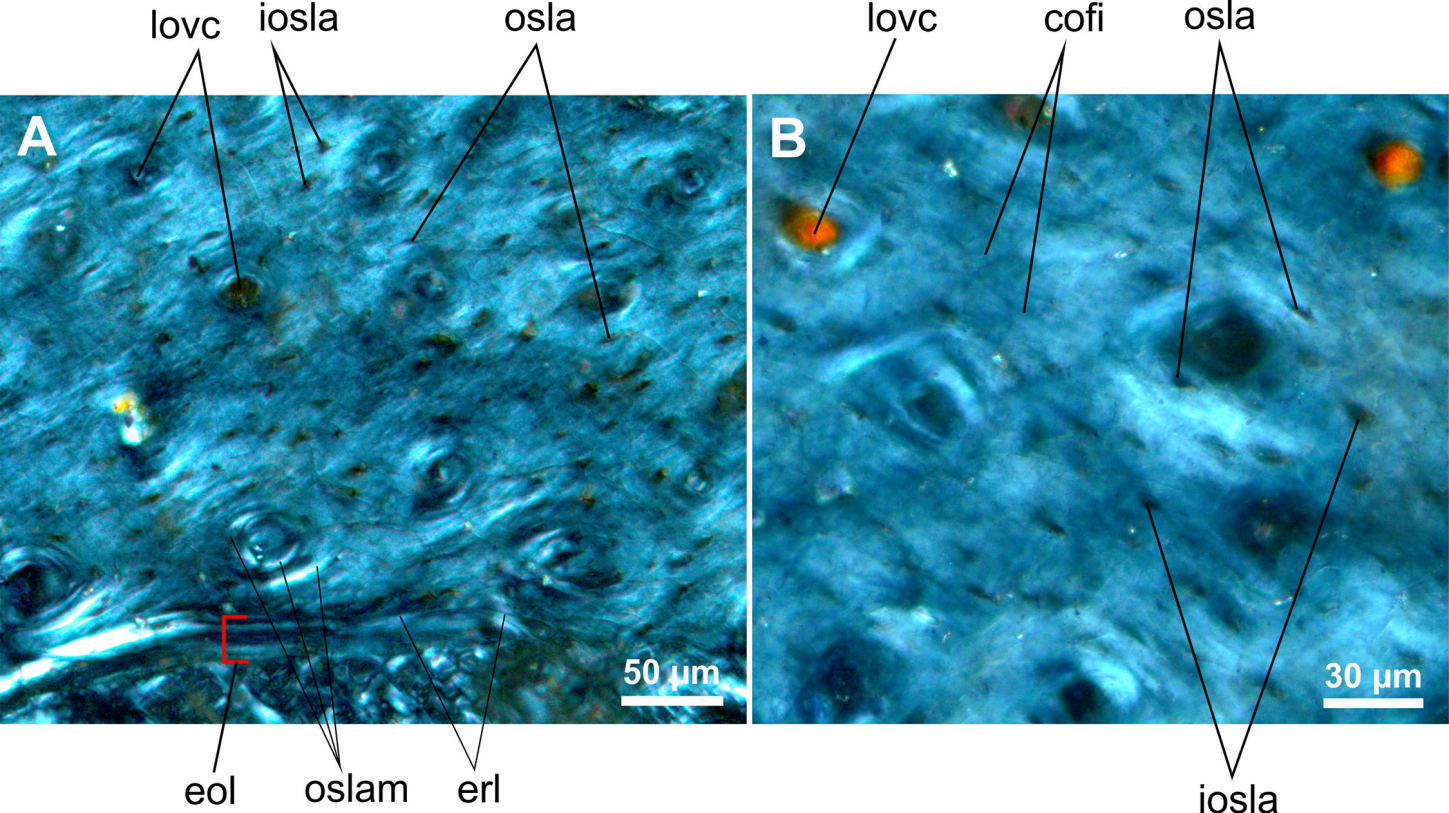
Transverse histological sections of the early juvenile tibia of *Gladocephaloideus jingangshanensis* (JPM 2014–004) viewed with polarized light microscopy. **A.** Close-up of the inner midshaft of the left tibia showing well-formed concentric centripetal lamellae of primary osteons; note laminated arrangement of the endosteal layer. **B.** Magnified view of the cortical bone containing bulky interosteonal lacunae; note that the osteonal lacunae are either oval or flat. Abbreviations: cofi, collagen fibres; eol, endosteal layer; erl, endosteal resorption line; iosla, interosteonal lacunae; lovc, longitudinal vascular canal; osla, osteonal lacuna; oslam, osteonal lamella.

JPM 2014–004 –mid-shaft of the left tibia (Figs 5 and 6): The periosteal cortex is approximately 730 to 770 microns thick. The cortex is well vascularized by numerous primary vascular canals that are mostly aligned with the longitudinal axis of the bone (Fig 5A). There are a few laminar and radial (anastomosing) vascular canals present as well. The primary osteonal canals have a diameter within the range of 15 to 28 microns at the outer cortex side, and 14 to 33 microns at the inner cortex side. We have observed a single secondary osteon in a deep region of the cortex. It is 120 x 165 microns across (Fig 5B), and the central canal, which is 47 microns in diameter, projects longitudinally. Neighboring this are two primary osteons with partly enlarged central canals suggesting an erosional osteoclastic activity in this region. Thus the thin cortex of the specimen is not entirely of primary bone. Some of the primary osteons are poorly defined and highly disparate in size. Based on our observations using polarized light, maximum diameter of a set of visible osteonal lamellae is roughly between 30 and 60 microns (Fig 6A). In fact, the concentric laminae are not always clearly visible and their varying number (? 3 to 5) is likely due to diagenetic alteration or/and an insufficient imaging technique used for this purpose. The bone matrix corresponds to the woven type and suggests that the bone had grown rapidly at this ontogenetic stage of the individual. Numerous canals in the outermost cortex open to the periosteal surface (Fig 5C). No LAGs or zones can be distinguished. The osteocyte lacunae are large and bulky or flattened (Fig 6B) and possess numerous radially projected lacunar canaliculi (Fig 5B), a feature usually attributed to an extensive transport of nutrients within the osteonal tissues. The innermost cortex is rimmed by an undulating erosional line that indicates defecting resorption of the oldest cortical tissue. The resorption is likely to balance the rapid growth of the primary cortex and the outward expansion of the medullary (marrow) cavity of the bone. A thin layer of bone tissue (maximum thickness: 22 microns) of endosteal origin was already deposited. The layer consists of at least five laminae visible in crossed polarized light. The medullary cavity appears to be free of trabeculae.

**Fig 7:**
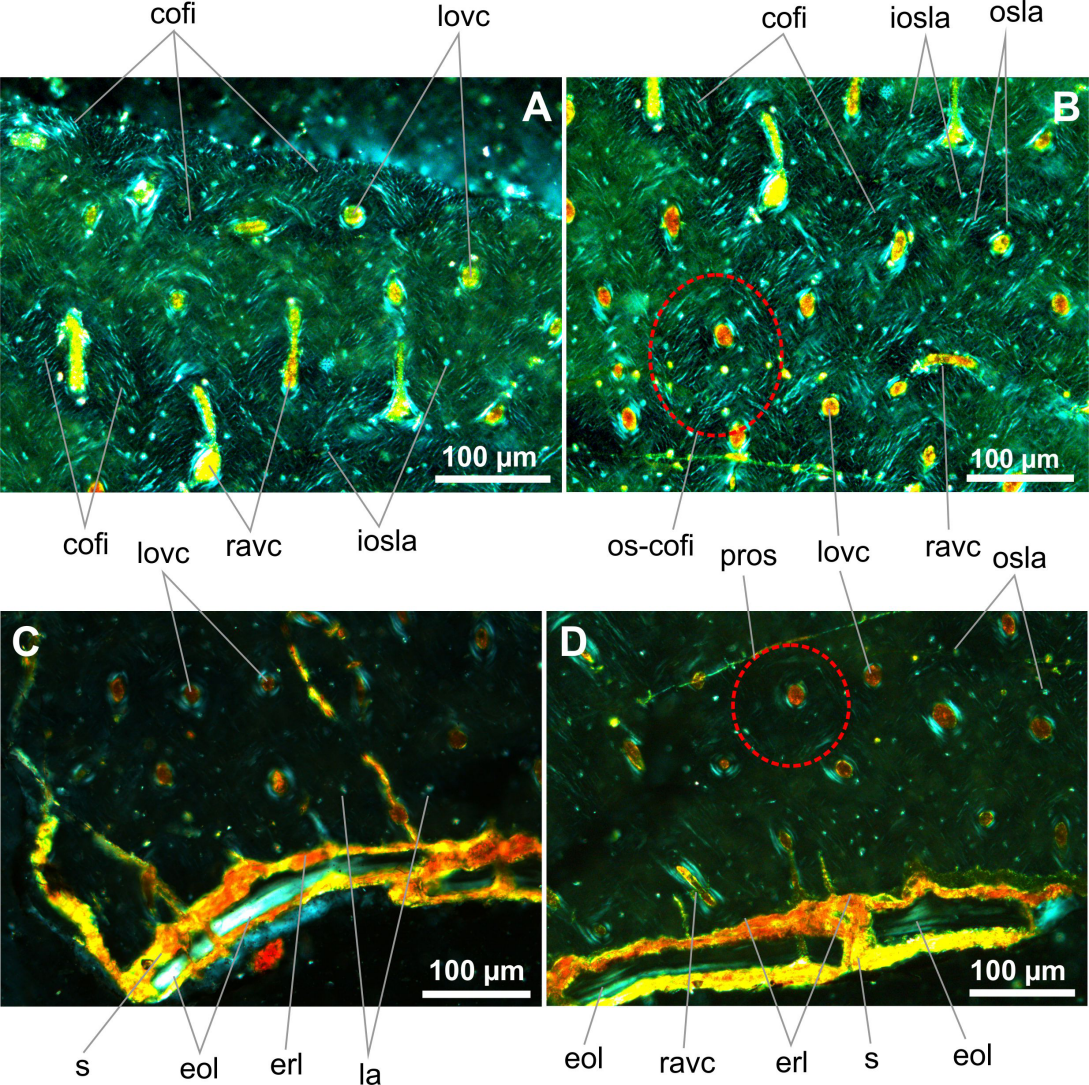
Transverse histological sections of the late juvenile tibia of *Gladocephaloideus jingangshanensis* (JPM 2014–004) viewed with transmitted light microscopy. **A.** The cortex of a lower midshaft of the left tibia; not a rich vascularization. **B.** Close-up of the outer midshaft dominated by vascular canals directed longitudinally and radially. **C.** Magnified view of the middle cortex showing bulky lacunae embedded in the woven bone matrix with randomly arranged collagen fibres. **D.** Close-up of the inner cortex containing longitudinal, laminar and radial vascular canals; note the innermost radial canal connecting the cortex with the medullary cavity. **E.** Magnified view of the cortex rimmed with a thicker endosteal lamina lacking any laminar texture; note eroded innermost surface of the cortex. Abbreviations: cofi, collagen fibres; eol, endosteal layer; erl, endosteal resorption line; ipovc; incomplete periosteal vascular canal; la, osteocyte lacuna; lavc, laminar vascular canal; lovc, longitudinal vascular canal; pros, primary osteon; po/me-vc, periosteal-medular vascular canal; povc, periosteal vascular canal; ravc, radial vascular canal.

**Fig 8:**
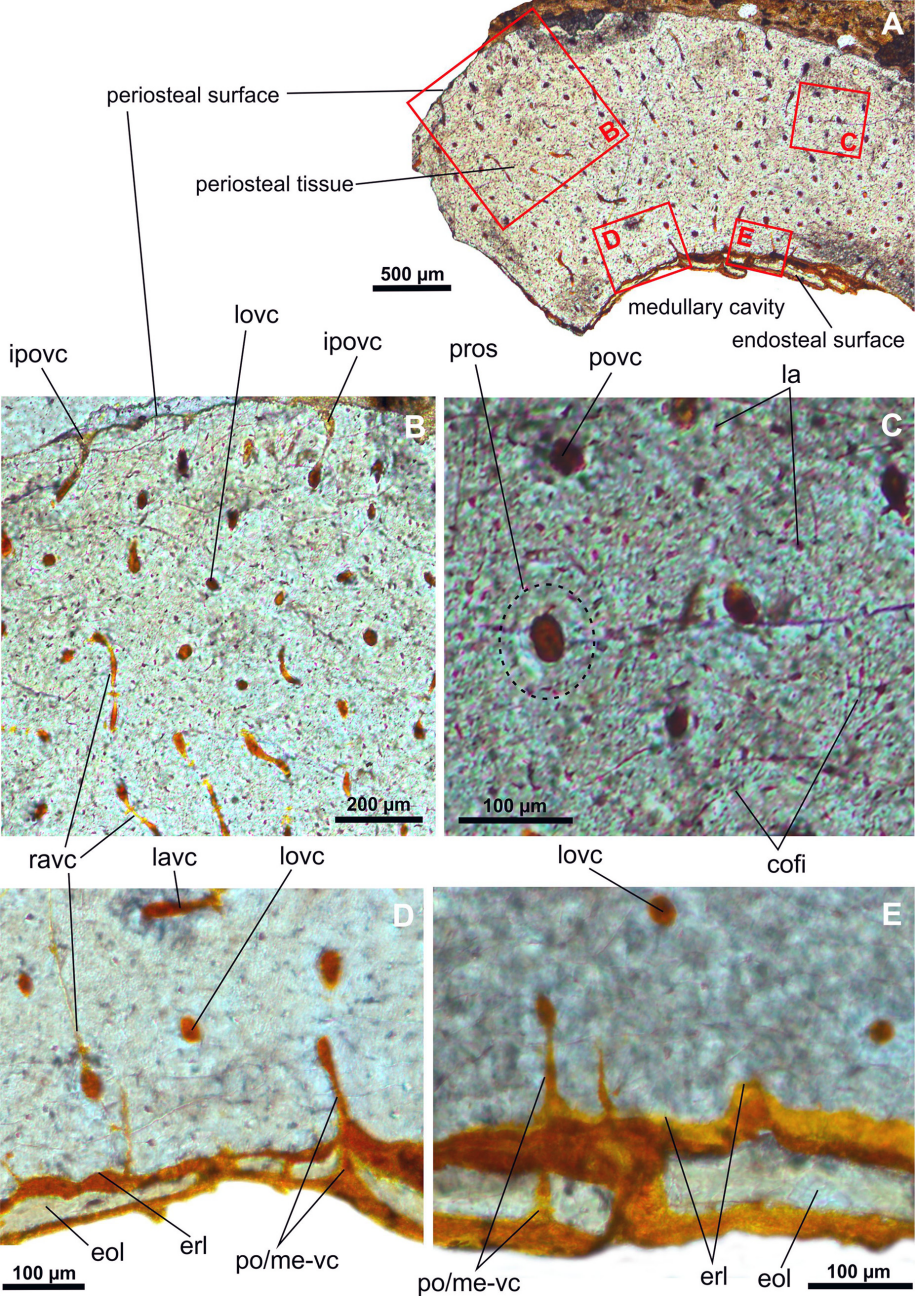
Transverse histological sections of the late juvenile tibia of *Gladocephaloideus jingangshanensis* (IG-CAGS 08–07) viewed with polarized light microscopy. **A.** Close-up of the outer cortex of the left tibia showing bundles of parallel and randomly distributed collagenous fibers. **B.** Close-up of the middle part of the cortex showing high density of collagenous fibers associated with the haversian bone; note relatively low number of interosteonal globular and ovoid lacunae; note flat osteonal lacunae. **C.** Close-up of the inner part of the cortex showing radial microfractures filled with sediment; note sediment depositions between the endosteal layer and the innermost cortical bone. **D.** Magnified view of the eroded innermost cortex and laminated endosteal layer. Abbreviations: cofi, collagen fibers; eol, endosteal layer; erl, endosteal resorption line; iosla, interosteonal lacuna; la, osteonal lamella; lovc, longitudinal vascular canal; os-cofi; osteonal collagen fibres; pros, primary osteon; ravc, radial vascular canal; s, sediment.

IG-CAGS-08-07 –lower part of the mid-shaft of the left tibia (Figs 7 and 8): The thickness of the periosteal cortex varies between 1340 and 1700 microns (Fig 7A). The periosteal tissue is densely vascularized through a network of longitudinal and radial canals (Fig 7B). Laminar vascular canals are also present, but they are not so frequent. Several canals are directed towards the periphery and are exposed on the outer surface of the bone. The periosteal cortex consists of mature osteonal units that are densely and irregularly distributed in the woven bone matrix and contain randomly oriented collagen fibers (Fig 7C). In cross polarized light, the most visible collagen fibers are arranged into bundles that are organized around and in parallel with osteonal canals, and project longitudinally and radially (Fig 8A and 8B). However, these organized fibers are crossed by other less apparent fibers at different angles but directed in more or less the same direction. Thus they form collagenous plexuses arranged radially or in parallel with the long axis of the bone rather than having a laminar pattern. No secondary osteon has been found in this bone sample. The primary osteonal canals are 18 to 35 microns in diameter whereas the osteons reach sizes of 60 to 75 microns. Osteocyte lacunae are bulky, mostly ovoid or globular (Fig 8C), but also flattened when situated between osteonal lamellae (Fig 8D). No zonal partition of the periosteal cortex by LAGs or annuli has been observed. The innermost cortex shows advanced erosional activity evidenced by the endosteal resorption line (Fig 7D and 7E). Some endosteal depositions had also taken place and formed a continuous endosteal layer that varies in its thickness (maximum thickness: 29 microns) (Fig 8C and 8D). The lamina itself is partly detached from the innermost cortex and separated by sediment. Furthermore, we assume that the endosteal layer was diagenetically altered in part as some of its microstructure can be discerned only locally (e.g. number of laminae: 5 to? 6), and some osteocyte lacunae, remained unrecognized. Several vascular canals cross the lamina suggesting that the endosteal bone was deposited around some blood vessels that connected the innermost cortex with the medullary cavity. No trabeculae are seen in the medullary space.

**Fig 9:**
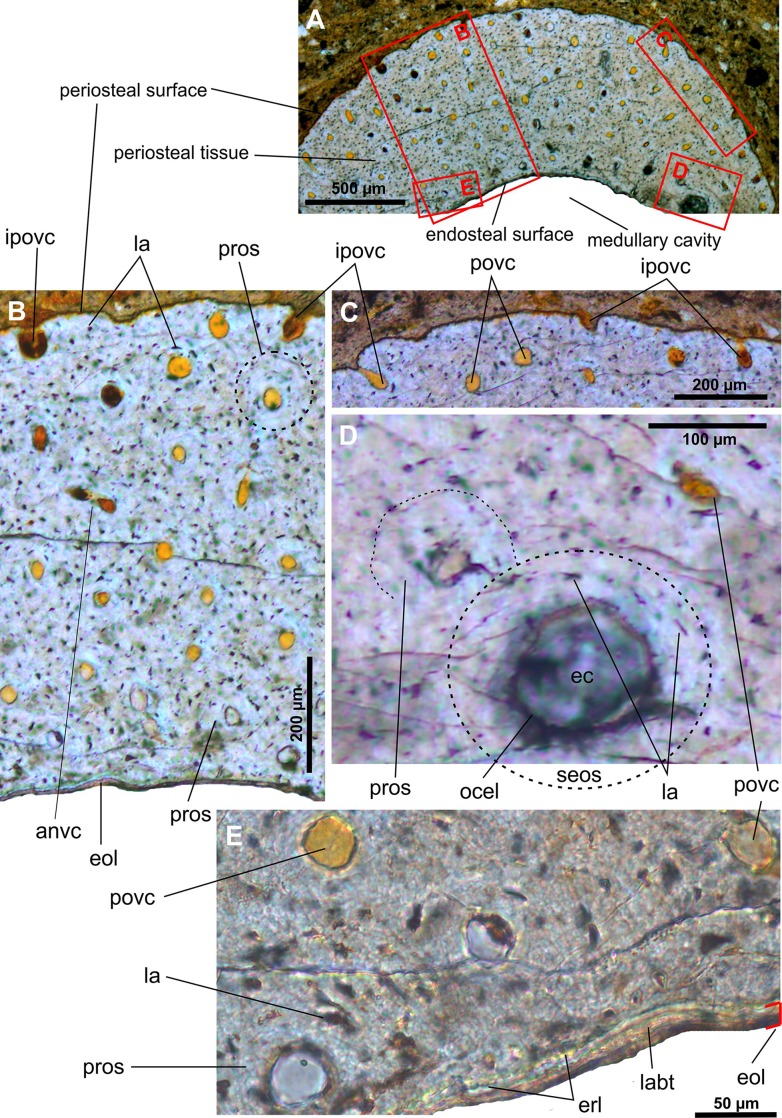
Transverse histological sections of the late juvenile tibia of *Gladocephaloideus jingangshanensis* (IG-CAGS 08–07) viewed with transmitted light microscopy. **A.** The thin cortex of an upper part of the midshaft of the right tibia. **B.** Close-up of the cortex profile dominated by high density of longitudinal, occasionally anastomosing, vascular canals embedded in the woven bone matrix. **C.** Close-up of the outermost cortex showing numerous vascular canals that open onto the periosteal surface. **D.** Magnified view of the eroded secondary osteon (note considerably flattened lacuane) and an initial erosion of the neighboring primary osteon. **E.** Magnified view of undulated and serrated innermost cortical surface documenting erosional activity; note laminated character of the endosteal bone. Abbreviations: anvc, anastomosing vascular canal; ec, erosional cavity; eol, endosteal layer; erl, endosteal resorption line; ipovc; incomplete periosteal vascular canal; la, osteocyte lacuna; labt, lamellar bone tissue; ocel, osteoclastic erosion line; pros, primary osteon; povc, periosteal vascular canal; pros, primary osteon; seos, secondary osteon.

**Fig 10:**
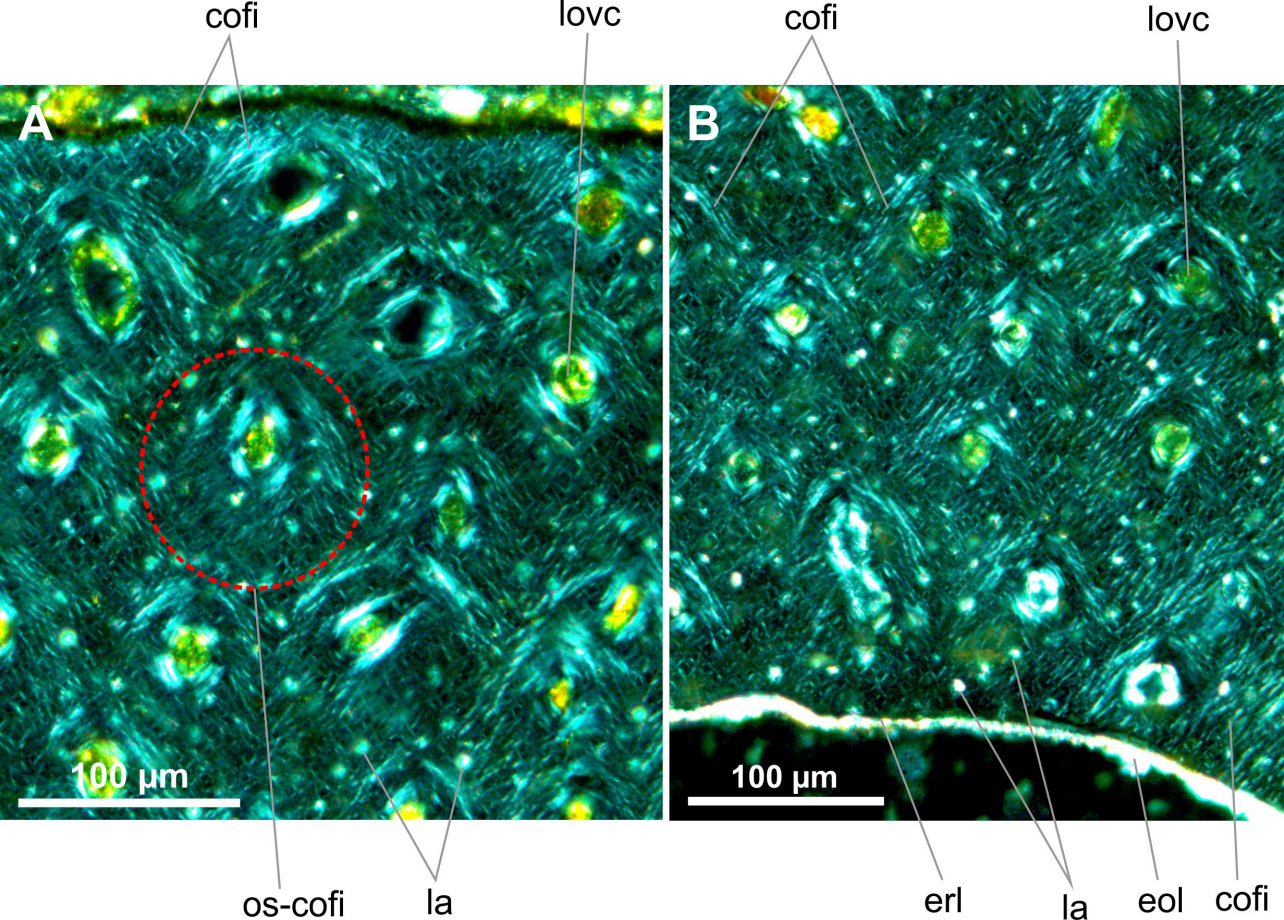
Transverse histological sections of the late juvenile tibia of *Gladocephaloideus jingangshanensis* (IG-CAGS 08–07) viewed with polarized light microscopy. **A.** Close-up of the outer cortex of an upper midshaft of the right tibia showing bundles of parallel and cruciate collagen fibers distributed around the longitudinal vascular canals; note relatively few bulky lacunae. **B.** Close-up of the inner cortex of the midshaft with radially oriented bundles of collagen fibers containing bulky lacunae; note irregular erosional line and thin endosteal bone. Abbreviations: cofi, collagen fibres; eol, endosteal layer; erl, endosteal resorption line; la, osteonal lamella; lovc, longitudinal vascular canal; os-cofi, osteonal collagen fibres.

IG-CAGS-08-07 –an upper (proximal) part of the diaphysis of the right tibia (Figs 9 and 10): The cortex is 760 to 850 microns thick (Fig 9A) and came from diaphyseal region that is closer to the end of the tibia than the previous sample. In general, the histology of the left and right tibia samples is quite similar. Both samples show a high density of vascular canals (with a wide range of diameters from 27 to 39 microns) distributed randomly throughout the cortex (Fig 9B). There are numerous incomplete vascular canals found at the outermost side of the bone (Fig 9C). The primary osteons (with diameters around 90–100 microns) are embedded in the woven bone matrix. The right tibia sample differs from the left tibia sample in several specific features: 1) dominance of longitudinal vascular canals (Fig 9D); 2) presence of secondary osteons; 3) higher osteoclastic activity shown by the presence of a large erosional cavity 98 x 114 microns; 4) deposition of thinner endosteal bone with a maximum thickness of 17 microns (Fig 9E); and 5) higher density of collagen fiber plexuses on the outer (centrifugal) side of the osteons (Fig 10A and 10B). There are a few osteonal lacunae that form quite large globular or ovoid structures with a maximum diameter up to 7.5 micron. No resting lines or any other growth marks have been recognized through the cortex. There are no endosteal trabeculae.

Both IG-CAGS-08-07 and JPM 2014–004 have thin-walled bones. The cortex of the tibia of the larger specimen (tibial length = 8.7 cm) is twice as thick as that of the smaller specimen (tibial length = 4.6 cm). As in other pterodactyloid pterosaurs, the periosteal bone of *Gladocephaloideus* is highly vascular. In contrast to most pterodactyloids in which the cortex consists of reticular fibrolamellar bone tissue [22–23], the periosteal bone of *Gladocephaloideus* is exclusively (in the smaller specimen) or largely (in the larger specimen) made of dense haversian bone containing a matrix of woven component. A single secondary osteons was found in the tibiae of both specimens, however the erosional cavity occurred only in the larger specimen, and developed inside of the secondary osteon. Similar erosional cavities have been recognized within the femoral cortex of an azhdarchoid pterodactyloid [24]. Bone redeposition had taken place in the deep region of the cortex of the larger specimen. The samples of tibiae show some traces of secondary resorption of the endosteal surface of the cortex. Furthermore, the endosteal surface was lined with an avascular laminar endosteal layer that is thinner in tibia of the smaller specimen.

Although lines of arrested growth are uncommon in pterydactyloids [23], there are some unambiguous LAGs preserved in the tibia of *Pterodaustro* [25], the metatarsal of a pterodactyloid and cranial bones of an ornithocheirid [23]. The bone samples of both specimens of Gladocephaloideus show that their cortices lack LAGs, annuli, zones or circumferential lamellae. This suggests that bone tissue grew rapidly without interruption during the juvenile growth stage of *Gladocephaloideus*. It is not clear whether JPM 2014–004 and IG-CAGS 08–07 represent developmental stages younger than one year, as the both specimens lack any growth line. Given size of the bones, it appears that IG-CAGS 08–07 is older than JPM 2014–004, however, the first growth line might have only appeared at somatic maturity, the condition likely not reach by either. Moreover, as some of the earliest periosteal tissue had been removed by endosteal resorption, it is possible that the first LAG was removed as well. We assume that both individuals underwent rapid periosteal deposition and had not attained skeletal maturity because the unfinished periosteal surface contains incomplete vascular canals. It was proposed that mature specimens of Pteranodon differ from juveniles in the poor vascularization near the outer periosteal surface [26]. Alternatively, the extent of endosteal remodeling and the presence of secondary osteons were used as an indicator of an adult stage of an azhdarchid pterosaur [27].

We suggest that the small *Glacocephaloideus jingangshanensis* specimen (JPM 2014–004) represents an earlier juvenile stagethan the holotype because the vascular canals that open onto the outer periosteal surface had not become complete osteons by the time the individual died. JPM-2014-004, however, is not a neonate due to presence of the secondary osteon in the cortex and the deposition of the endosteal layer. The larger specimen holotype (IG-CAGS 08–07) had been identified as an adult individual [11]. Our study indicates that the bones of IG-CAGS 08–07 still show clear signs of active growth: absence of the external fundamental system, LAGs, cortical zonation, and substantial bone remodelling. We conclude that IG-CAGS 08–07 is not a fully grown individual and its bone samples may document the late juvenile to subadult stage of skeletal development of *Glacocephaloideus* ontogeny.

## Conclusion

**Fig 11:**
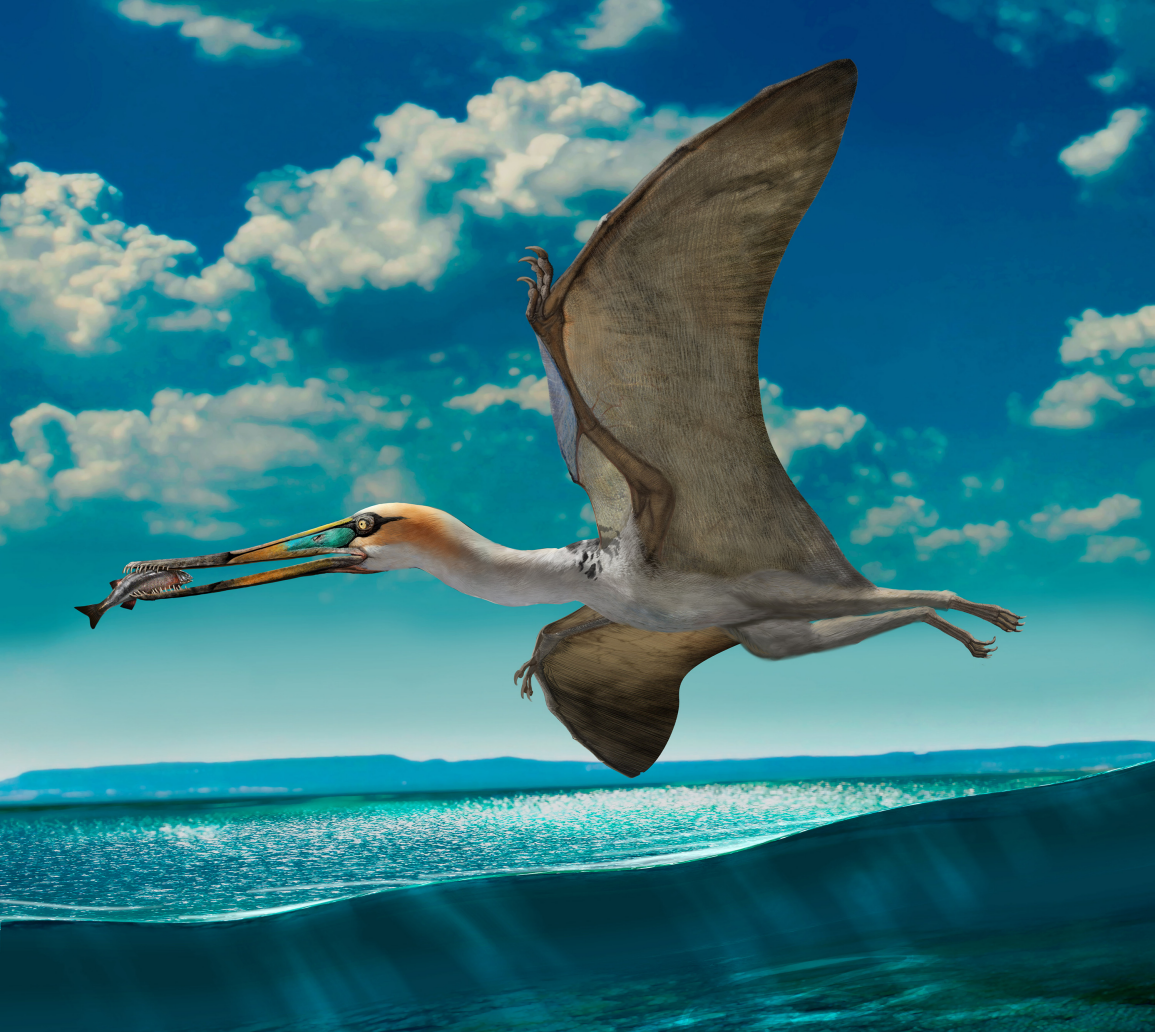
Life reconstruction of *Gladocephaloideus jingangshanensis* (drawn by Zhao Chuang).

The new material of *Gladocephaloideus* provides much more information on the postcranial morphology, such as the length ratios of femur to tibia, length-to-width ratio of the cervical vertebra, and the length relationships of wing phalanges and tibia. The dense, slender, sharp teeth suggest that *Gladocephaloideus* is a fish eater (Fig 11). Phylogenetic analysis shows that *Gladocephaloideus* is more closely related to *Pterofiltrus* than to other ctenochasmatoids, and they form a clade. The description of a new specimen of *Gladocephaloideus* from the Jiufotang Formation and the holotype IG-CAGS 08–07 from the Yixian Formation substantially expand the geographic range of this taxon, and increase the known diversity of the pterosaur assemblage from this area. Therefore, the discovery of new material of *Gladocephaloideus* indicates that the toothed pterosaurs from western Liaoning are widely distributed and more diverse than previous thought. Bone microstructure indicates that JPM 2014–004 represents an early juvenile stage of *Gladocephaloideus jingangshanensis* and the holotype, IG-CAGS-08-07, is also not fully mature. The holotype bone tissues document an initial stage of skeletal maturation equivalentto a late juvenile or sub-adult stage.

## Supporting Information

S1 File The file contains measurements and phylogenetic analysis. (DOC)
