# Supplementary material for: New Material of the Pterosaur Gladocephaloideus Lü et al., 2012 from the Early Cretaceous of Liaoning Province, China, with Comments on Its Systematic Position
Source: PLoS One. 2016 Jun 1;11(6):e0154888. doi: 10.1371/journal.pone.0154888 (PMC4889066; doi:10.1371/journal.pone.0154888)
Supplement: S1 File — (DOC) [file pone.0154888.s001.doc]

# Supporting Information for:

New material of the pterosaur *Gladocephaloideus* Lü et al., 2012 from the Early Cretaceous of Liaoning Province, China, with comments on its systematic position

Junchang Lü^1^, Martin Kundrát^2^, and Caizhi Shen^1^

1. Institute of Geology, Chinese Academy of Geological Sciences, Beijing100037, China; Key Laboratory of Stratigraphy and Paleontology, Ministry of Land and Resources; E-mail address: [Lujc2008@126.com](mailto:Lujc2008@126.com); Tel. (fax). 00861068999707

2. Department of Ecology, Faculty of Natural Sciences, Comenius University, SK-84215 Bratislava, Slovak Republic

* To whom correspondence should be addressed. E-mail: [lujc2008@126.com](mailto:lujc2008@126.com) (Lü, J.-C.)

CONTENTS:

1. Measurements
2. Phylogenetic analysis
   - 1. Analysis Protocol
     2. Character List
     3. Data Matrix

1. Measurements

Table S1. Measurements of *Gladocephaloideus jingangshanensis* (JPM-2014-004) in mm

| Element | Length | Width |
| --- | --- | --- |
| Skull | 108.8 | - |
| Orbital diameter | - | 10.9 |
| Nasoantorbital opening | 14.1 | - |
| Prenarial length | 68.5 |  |
| Lower jaw | 87.9 | - |
| Mandibular symphysis | - | 2.8 |
| Cervical series | 73.3 |  |
| 2^nd^ to sixth cervical vertebrae | 14.0/17.4/16.4/15.2/12.9 | 4.1/4.2/4.7/4.9/4.8 |
| Coracoid | 19.8 | 3.2 |
| Scapula | 21.9 | 3.5 |
| Humerus | 30.0 | 4.0 |
| Ulna/radius | 39.1/40.0 | 2.8/2.1 |
| Pteroid | 16.8 | 0.8 |
| Metacarpal IV | 32.4 | 2.8 |
| Metacarpals I, II | 29.2/27.0 | 0.6/0.4 |
| wing phalanges 1-4 | 45.5/42.1/37.5/33.3 | 2.5/2.4/1.7/1.3 |
| Femur | 27.5 | 3.8 |
| Tibia | 45.0 | 2.2 |
| Metatarsals I-IV | 17.8/17.8/18.6/16.8 | 0.8/0.7/0.5/0.6 |
| Pes | 28.2 | 6.5 |

B. Phylogenetic analysis

1. Analysis protocol

In order to determine the systematic position of *Gladocephaloideus*, a phylogenetic analysis was conducted based on the data matrix of Lü et al. [S1] with the addition of 118characters coded for *Gladocephaloideus* and *Guidraco*. The character coding of *Gladocephaloideus* is based on both the holotype *Gladocephaloideus* and the new specimen (JPM 2014-004). The data matrix consists of 67 ingroup taxa and 118 characters.

The analysis is performed by TNT v1.1 [S2] using a ‘new technology’ search (with default parameters for sectorial search, ratchet, tree drift, and tree fusion), which recovered a minimum length tree in 10 replicates. This procedure aims to broadly sample tree space and identify individual tree islands. We then subjected the recovered most parsimonious trees (MPTs) to a traditional search with TBR branch swapping, which more fully explores the tree islands found in the ‘new technology’ search. This process returned a total of 1296 MPTs of 466 steps (consistency index=0.356, retention index=0.761). Supplementary Figure 1 is the strict consensus of 1296 most parsimonious trees with common synapomorphies.

In all trees, the clade Monofenestrata is a monophyletic group and is supported by the following synapomorphies: rostral index is greater than 3.0 (character 6, state 2); nasal process of maxilla is absent (character 13, state 2); maxilla-nasal contact is lost (character 14, state 2); nares is smaller than the orbit or nasoantorbital opening (character 17, state 1); naris and antorbital opening is confluent (character 20, state 1); unguals of manus and pes are similar in size (character 95, state 0); distal end of pteroid: tapering or pointed (character 118, state 1).

The clade Pterodactyloidea is supported by the following synapomorphies: cervical ribs is highly reduced or absent (character 66, state 1); number of caudal vertebrae is 15 or fewer (character 70, state 1); combined length of caudal vertebrae is shorter than the dorsal series (character 71, state 1); filiform extensions of zygapophyses and hypapophyses is absent (character 72, state 0); ulna/tibia ratio is smaller than 0.9 (character 89, state 3); fifth pedal digit bears one very short phalange, or less (character 116, state1). Archaeopterodactyloidea is supported by the following synapomorphies: posterior region of skull is rounded (character 29, state 1); squamosal position is entirely below the orbit (character 30, state 1); occiput faces ventrally (character 32, state 1); quadrate is subhorizontal (character 34, state 2); lateral pneumatic foramen on centrum of the cervical is present (character 64, state 1); and anterior profile, in lateral view, of pubis is slightly concave (character 104, state 1). Ornithocheiroidea is supported by the following synapomorphies: postexapophyses are present on cervical vertebrae (character 63, state 1); ulna is similar in length to dorsals+sacrals (character 87, state 1) and only I and IV contact syncarpal between distal syncarpal and metacarpals I-IV (character 93, state 1). Pteranodontia is supported by the following synapomorphies: preorbital rostrum is greater than 80% skull length (character 7, state 1). posterior margin of nasoantorbital fenestra is concave (character 22, state 1); basal region of orbit is infilled (character 25, state 1); notarium is present (character 69, state 1); proximal surface of scapula is sub-oval (Character 78,, state 1); diameter of radius is less than half the ulna (character 86, state 1); ulna/tibia ratio is less than 0.9 (character 89, state 3); ornithocheiroid carpus is present (character 90, state 1); unguals of manus and pes are twice the size, or more, of pedal unguals (character 95, state 1); manus digit iv (wing-finger) phalange 1 compared to length of tibiotarsus is 1.5-2.0 times longer (character 98, state 2); femur caput directed steeply almost parallel to long axis of femur shaft (character 108, state 1) and fibula reduced to a small splint or lost altogether (character 113, state 2). Ctenochasmatoidea is supported by the following three synapomorphies: mid-series cervical is elongate (character 65, state 1); neural arch of cervical are depressed down onto, or even confluent with, the centrum (character 67, state 1) and neural spines of mid-series cervical are low or absent (character 68, state 2). Ctenochasmatidae is supported by the following two synapomorphies: prenarial rostrum is more than 50% skull length (character 5, state 1) and teeth are relatively elongate with a long cylindrical crown of even width and a short tapering distal tip (character 60, state 1). Azhdarchoidea is supported by the following three synapomorphies: caudal end of mandible with distinct dorsal 'coronoid' eminence (character 47, state 0); heterodonty in the mandibular dentition is absent (character 54, state 1); leg length (Femur+Tibia) is more than 1.5 times the length of dorsal+sacral vertebral series (character 107, state 1); prominent anteriorly directed tubercle is present on dorsal apex of external trochanter of femur (character 110, state1) and prominent anteriorly directed tubercle is present on dorsal apex of external trochanter of femur (character 112, state 1).


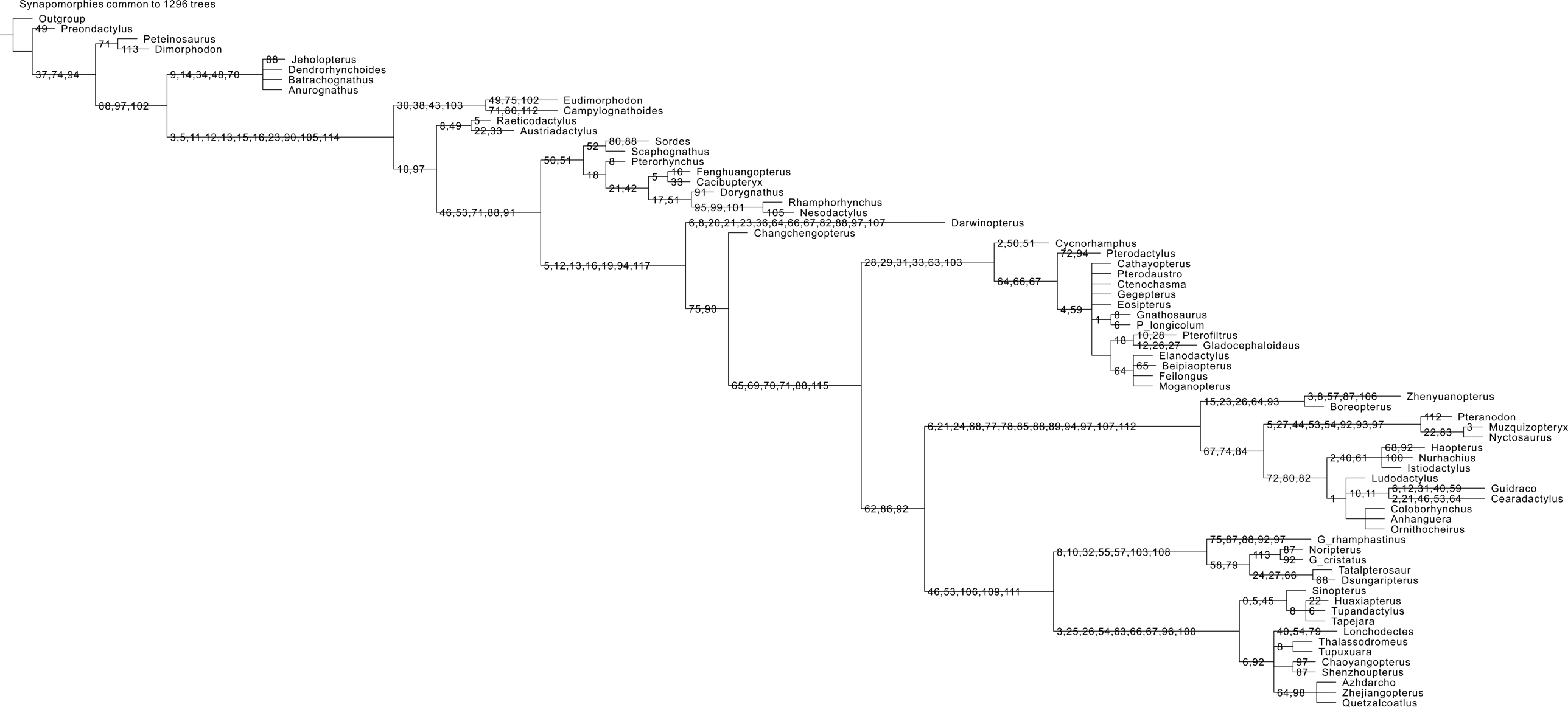


Fig. S1. The strict consensus of 1296 most parsimonious trees, with distributions of common synapomorphies.

2. Character List

The character list and data matrix based on [S1] with the addition of *Gladocephaloideus*, *Guidraco*, *Beipiaopterus*, *Elanodactylus* and *Pterofiltrus*.

1. Tip of rostrum downturned: absent (0); present (1).

2. Tip of rostrum laterally expanded: absent (0); present (1).

3. Tip of rostrum: laterally compressed (0); dorsoventrally compressed (1).

4. Rostrum: high with convex outline (0); low with straight or concave dorsal outline (1); anterior region of rostrum low, but antorbital region expanded dorsally (2).

5. Prenarial rostrum: less than (0); or more than 50% skull length (1).

6. Rostral index: 1.5 or less (0); 1.5-3.0 (1), >3.0 (2).

7. Preorbital rostrum: <80% skull length (0); >80% (1).

8. Dorsal margin of nasoantorbital opening bounded by slender bar: absent (0); present (1).

9. Premaxillary crest: absent (0); low, rounded, confined to rostrum (1); comb-like free margin and extends from above anterior end of nasoantorbital fenestra to apex of skull (2); extends from tip of rostrum to apex of skull and confluent with fronto-parietal crest (3); tall, narrow, stands on anterior half of premaxillae (4); keel-like, anterior margin extends no further forward than midpoint of nasoantorbital fenestra (5).

10. Skull broad with very short preorbital region: absent (0); present (1).

11. Ventral margin of skull: straight (0); downcurved caudally (1).

12. Posterior extent of premaxillae: terminates level with frontals (0); overlaps frontals (1).

13. Nasal process of maxilla: vertical-subvertical (0); inclined backwards (1); absent (2).

14. Maxilla-nasal contact: narrow (0); broad (1); lost (2).

15. Nasal opening: terminal (0); subterminal (1).

16. External nasal opening: height similar to or greater than anteroposterior length (0); elongate (1).

17. Nares: form the largest skull opening (0); smaller than the orbit or nasoantorbital opening (1).

18. Antorbital fenestra lies behind and below the naris: absent (0); present (1).

19. Antorbital fenestra: less than twice as long as it is deep (0); at least as twice as long as it is deep (1).

20. Naris and antorbital opening: separate (0); confluent (1).

21. Nasoantorbital fenestra: <40% skull length from tip of rostrum to posterior margin of orbit (0); >40% (1).

22. Posterior margin of nasoantorbital fenestra: straight (0); concave (1).

23. Orbit shape: subcircular, jugal processes at right angle (0); tall, oval, jugal processes at acute angle (1).

24. Orbit: smaller than antorbital opening (0); larger (1).

25. Basal region of orbit: open (0), infilled (1), infilled by horizontal bar (2).

26. Dorsal margin of orbit: level with or above dorsal margin of nasoantorbital opening (0); or below it (1). [modified, based on the skull of *Gladocephaloideus*. The dorsal margin of orbit is above dorsal margin of nasoantorbital opening in *Gladocephaloideus*]

27. Frontal extends anterior to the lacrimal-jugal bar: absent (0); present (1).

28. Fronto-parietal crest: absent (0); flange-like, short (1); flange-like, elongate (2); rod-like, short (3); rod-like, elongate (4); sail-like (5).

29. Posterior region of skull rounded: absent (0); present (1).

30. Squamosal position: above, or level with the orbit (0); entirely below the orbit (1).

31. Supratemporal fenestra largest skull opening after the orbit: absent (0); present (1).

32. Occiput: faces posteriorly or posteroventrally (0); or ventrally (1).

33. Distal ends of paroccipital processes: unexpanded (0); rounded, tongue-like flange (1).

34. Quadrate: vertical (0); inclined backward (1); subhorizontal (2).

35. Palatal elements reduced to thin bars of bone: absent (0); present (1).

36. Position of the jaw joint: under the posterior third of the orbit (0); under the middle third of the orbit (1); under the anterior third of the orbit (2).

37. Palatal ridge: absent (0); present (1).

38. Dentary: <50% length of lower jaw (0); >50% (1).

39. Anterior tip of the mandible: horizontal (0); downturned (1).

40. Anterior end of the lower jaw: unexpanded (0); expanded (1).

41. Anterior end of mandibular symphysis: laterally compressed or as wide as deep (0); dorsoventrally compressed (1).

42. Bony mandibular symphysis: absent (0); present (1).

43. Mandible tips fused into a short symphysis bearing a forward projecting 'tooth-like' prow and a number of large, fang-like, procumbent teeth forming a fish grab: absent (0) present (1).

44. Anterior end of dentary: level (0); dorsally expanded forming low rounded eminence (1); high rounded eminence (2).

45. Mandibular rami; level with symphysis (0); elevated well above level of symphysis (1).

46. Dentary bony sagittal crest: absent (0); present (1).

47. Caudal end of mandible with distinct dorsal 'coronoid' eminence: present (0); absent (1).

48. Dimorphodontid dentition: absent (0); present (1).

49. Teeth are small, peg-like and widely spaced: absent (0); present (1).

50. Multicusped teeth: absent (0); present (1).

51. Rostral dentition: more than 11 pairs of teeth (0); less (1).

52. Rostral dentition: more than nine (0); nine or less relatively straight (or slightly recurved teeth (1).

53. Mandibular dentition: more than six pairs of teeth (0); six or less (1).

54. Heterodonty in the mandibular dentition: present (0); absent (1).

55. Dentition: present (0); absent (1).

56. Largest teeth in caudal half of dentition: absent (0); present (1).

57. First three pairs of teeth large, 4th-6th small, 7th-9th large: absent (0); present (1).

58. Short, broad teeth in at least part of the dentition: absent (0); present (1).

59. Dentition: extends to jaw tips (0); jaw tips toothless, but followed by tooth row (1).

60. Teeth relatively elongate with a long cylindrical crown of even width and a short tapering distal tip: absent (0); present (1).

61. Total of more than 192 long, fine teeth: absent (0); present (1).

62. Laterally compressed, triangular teeth in at least part of the dentition: absent (0); present (1).

63. Postexapophyses on cervical vertebrae: absent (0); present (1).

64. Lateral pneumatic foramen on centrum of the cervical: absent (0); present (1).

65. Mid-series cervicals: short (0); elongate (1); very elongate (2).

66. Cervical ribs: present (0); highly reduced or absent (1).

67. Neural arch of cervicals: high (0); depressed down onto, or even confluent with, the centrum (1).

68. Neural spines of mid-series cervicals: tall, blade-like (0); tall, spike-like (1); low or absent (2).

69. Notarium: absent (0); present (1).

70. Number of caudal vertebrae: more than 15 (0); 15 or fewer (1).

71. Combined length of caudal vertebrae: longer than the dorsal series (0); shorter (1).

72. Filiform extensions of zygapophyses and hypapophyses: absent (0); present (1).

73. Sternum: absent (0); rectangular (1); triangular (2); semicircular (3), square with posterolateral projections (4).

74. Cristospine of sternum: unconstricted (0); constricted (1).

75. Coracoid: less than two thirds length of scapula (0); from at least two thirds up to similar length to scapula (1); longer than scapula (2).

76. Coracoid with well-developed brachial flange: absent (0); present (1).

77. Coracoidal contact surface with sternum: articulation surface flattened, lacking posterior expansion (0); articulation surface oval, with posterior expansion (1).

78. Proximal surface of scapula: elongated (0); sub-oval (1).

79. Shape of scapula: elongate (0); stout with constricted shaft (1).

80. Appendicular bones with thin cortex and wide lumen: absent (0); present (1).

81. Forelimb: up to 2.5 times length of hind limb (f+t+mt) (0); 2.5-3 times length of hind limb (1); 3-4 times length of hind limb (2); at least 4 times length hind limb (3).

82. Pneumatic opening in palmar surface of humerus: absent (0); present (1).

83. Pneumatic opening in anconal surface of humerus: absent (0); present (1)

84. Deltopectoral crest of humerus: small (0); large and subtriangular with apex directed proximally (1); proximo-distally elongate, rectangular (2); hatchet-shaped (3); tongue-shaped with necked base (4); antero-posteriorly elongate with rectangular shape (5); warped (6); distally expanded (7).

85. Distal end of humerus: D-shaped (0); triangular (1).

86. Diameter of radius: more than half the ulna (0); less (1).

87. Ulna: considerably shorter than dorsals+sacrals (0); similar in length to dorsals+sacrals (1).

88. Ulna: less than 133% humerus (0); 133-150% (1); >150% (2).

89. Ulna/tibia ratio: 0.9-1.2 (0), 1.2-1.4 (1); >1.4 (2); <0.9 (3).

90. Ornithocheiroid carpus: absent (0); present (1).

91. Pteroid: less than 30% length humerus: (0); 30-60% (1); >60% (2).

92. Metacarpals I-III: disparate lengths (0); the same length (1).

93. Contact between distal syncarpal and metacarpals I-IV: all four in contact (0); only I and IV contact syncarpal (1); only IV contacts the syncarpal (2).

94. Wing-metacarpal: humerus ratio: less than 0.8 (0); between 0.8 and 2.2 (1); > 2.2 (2).

95. Unguals of manus and pes: similar in size (0); manual unguals twice the size, or more, of pedal unguals (1).

96. Manus digit iv (wing-finger): 57.5%, or less, of total forelimb length (0): >57.5% (1); >65% (2).

97. Proceeding distally, wing phalanges 1-4 exhibit a rapid decline in length, contributing 40%, 30%, 20% and 10% to the wing-finger respectively: absent (0); present (1).

98. Manus digit iv (wing-finger) phalange 1 compared to length of tibiotarsus: shorter (0); 1 to 1.5 times longer (1); 1.5-2.0 times longer (2); more than twice the length (3).

99. Longitudinal ventral ridge on wing-phalanges two and three: absent (0); present (1).

100. Wing-finger phalanges with deep posterior groove: absent (0); present (1).

101. Contribution of wing-phalange 1 to wing-finger length: less than 30% (0); 30-40% (1); more than 40% (2).

102. Manus digit iv (wing-finger) phalanges: decline in length distally (0); phalanges 2 and/or 3 longer than phalange 1 (1).

103. Preacetabular process of ilium: similar length to postacetabular process (0); longer (1).

104. Anterior profile, in lateral view, of pubis: convex or straight (0); slightly concave (1); deeply concave (2).

105. Pubis and ischium: unfused (0); fused to form a plate with a straight ventral margin that meets the posterodorsal margin at an acute angle (1); with convex ventral border, ischium that projects below level of the pubis and obtuse posterior apex (2); dsungaripterid ischiopubis (3).

106. Prepubis: distal expansion longer than broad or similar width to length (0); transversely expanded (1); co-joined prepubes forming H shape (2).

107. Leg length (Femur+Tibia): less than 1.5 x length of dorsal+sacral vertebral series (0); more than 1.5 times (1).

108. Femur caput: directed inward at about 135° (0); directed steeply almost parallel to long axis of femur shaft (1).

109. Strongly bowed femur: absent (0); present (1).

110. Prominent anteriorly directed tubercle on dorsal apex of external trochanter of femur: absent (0); present (1).

111. Pneumatic opening in posterior face of collum femoris: absent (0), present (1).

112. Prominent anteriorly directed tubercle on dorsal apex of external trochanter of femur: absent (0); present (1) (1).

113. Fibula: subequal in length (0), or less than 80 percent the length of the tibia (1); reduced to a small splint or lost altogether (2).

114. Length of metatarsal III compared to tibia: <30% (0); >30% (1).

115. Length of metatarsal 4: similar in length of metatarsals i-iii (0); shorter than metatarsals i-iii (1).

116. Fifth pedal digit: two phalanges (0); one very short phalange, or less (1).

117. Phalange two of pedal digit v with distinctive angular flexure at mid-length, such that the distal half of the phalanx lies at 40-45° to the proximal half: absent (0); present (1).

118. Distal end of pteroid: expanded, and knob-like (0); tapering or pointed (1) [new character]

3. Data Matrix for phylogenetic analysis

Based on the new information of *Cearadactylus* (S2), the codings for some characters are changed as follows: 9(1); 11(0); 12 (0); 16(1); 17(0); 19(0); 21(0); 23(1); 37(1); 41(0); 47(0); 57 (1); 60(0)

Outgroup 000{01}000000000000{01}0000000000000000000?000000000000000000000000000000000000?0000000000000000000000000000000?0{01}000000000?

*Preondactylus* 0000000000?0001000000?10000000?00??0?0000000000101000000000000??0000?000??00?0010??1?00000000101000001001?0000??1100?0

*Dimorphodon* 000000000000001000000010000000000000?100000000010000000000000001000?0001??1000011001000{01}000001110000010010000000000000

*Peteinosaurus*

???????0?00??????????????????????????1000000000100000000000000??0?0?0001?01000011?010000000001110000010?10000??0010000

*Anurognathus*  00?000000100000000000?0000000000??10?100000000?0101101000000000?010?0010??10?001100200111000001101002010??000000110000

*Batrachognathus* 00?000000100000000000?00000000?00?10?1000000000010000100000000??0???0010101?0001??0200??1??0??1???????1?????00??110000

*Dendrorhynchoides* 00?000?001??0000??0?0??????00??0??1????00?0000?010????000000000?01000010??1000012?0200101000001102?0201???0100?0110000

*Jeholopterus* 00?0000001????0????00?????000??0???0???00?0000?010????00000000??01?20010??1000012?020011200?00110200101??00000?0110000

*Austriadactylus* 0001010020?1111110000011000000?0?0?0?1?00???0?0001000000000000??00000000?????001????00???0????1?0?00?1??????0???1?????

*Campylognathoides* 000101000001111110000001000000100100?11000010000000000000000000100000001401000013003000010{01}001120200011121000000011000

*Eudimorphodon* 000101000001111110000001000000100100?11000010000010000000000000?00000000401100012003000{01}{01}010011{12}0{12}000101210{01}0000111000

*Raeticodactylus* 00010000201?11111000000100?000000100?1000?02010001000000000000010?000??????????1?0?30??01??0????0100?1?????000001?????

*Scaphognathus* 00010100001111111000000100000000010001000100001000111100000000??000000012010?001200400022011011101000110210000?0111010

*Sordes*  000101000011111110000001000000000100?1000100001000111100000000??000000012?100001300400021011011101000110210000001{01}1010

*Pterorhynchus*  0001010020111111101000010000000001?0?1000?00001000110100000000??0?00?001???????12???00022?1?01?1010001????0?0??????0??

*Cacibupteryx* 00010000001111111010011100000000?0000??????????00?1?0?000???0?????????????????????????????????????????????????????????

*Dorygnathus* 000101000011111111100111000000000100?1000110001000100100000000??000000012010000120040002201001110100011021000000111000

*Nesodactylus*

???????????????????00???????????????????????????????????????????0000???120100001?00400?2?0?101?20?01001020?0??????????

*Rhamphorhynchus*  000102000011111111100101000000000100010001100010001001000000000100000001{12}010000130040002201101120301001021000000111000

*Darwinopterus* 00010210201122110001111000000000010111000100001000000100000000??10120001301000012?11000{12}001101010000011?210100?0111011

*Pterodactylus* 000102000011221100010001000011010201010001000010000001000000000011120110201000012005000{01}002102110{01}00{01}011210000001111?1

*Cycnorhamphus* 001102000011221??0?1000?0000110102?201001100001000110100000000000100011030110001200500013021020101001011210000?0101???

*Eosipterus*

????????????????????????????????????????????????????????????????11120110??11000110050000001102010100001???0000001111?1

*P_longicolum* 011112000011221??0?10?0?00001?010201010111000010000001000001001?11120110??1100010005000030?1120001001011211000?01011??

*Gnathosaurus*  0111121020112211?001000100001101020101011100001000000100000100????????????????????????????????????????????????????????

*Cearadactylus* 01111210100022110001001?10?????0?10211010100000000000000100000111110??????????????????????????????????????????????????

*Gegepterus* 0011121000112211?0010001000011010201?10?110?0010000001000001101111120??????10??1?????????0??????0100?01?????0??011???1

*Ctenochasma* 00111210{02}011221??001000?000011010201?1001100001000000100000110??11120110?01?00011?05000{01}0?1102010100101?210000001111?1

*Pterodaustro*  001112100011221??001000?000011010201?1001100001000000100000110111112?01030110001000500?000?1120101001011210000001111?1

*Istiodactylus* 001101000011221??001101?000000000102010011000010000001000000011101011????0200111301611?211?012?1020010?????100002????1

*Nurhachius* 0011021000?122110001101100?0??0??1020100110000100000010?000001?101011?????200111?0?6?1?2???112?10200201????100??201??1

*Haopterus*  001101?000??221??0010??????0?????10??1001100001000000100100001??010?0???3020?111???611?1?11002110?0010????????????11?1

*Ornithocheirus*  010102101011221??0?1011?10010000010211010100011000000100100000????????????????????????????????????????????????????????

*Anhanguera* 010102101011221100010111100000000102110101000{01}10000001001000001101011110312011113016111{12}11211211020010101?11000020????

*Coloborhynchus* 010102101011221??0?1011?100000000102110101000110000001001000001101011110312011113016111111?11211020010101?0100002011??

*Ludodactylus* 010102100011221100010111100100000102?10?0100001000000100100000????????????????????????????????????????????????????????

*Boreopterus* 000112100011221000010110101000?001?0?10001000010000001001000001?1100?110???????111???1111?111111020010????0100??201101

*Nyctosaurus* 000113100011221??0?1010?100200000102010001001010000000100000001001011110102101112107?112212?23?{01}03002011120100002111?1

*Muzquizopteryx*

???0???00011221??0?1?10?1002000001?2?1??????101000000010000000??010?111011?1??11??07?11?????2?????????1??20100002??1?1

*Pteranodon*  000113100011221??0?1011?10020000010201000100101000000010000000110101111011200111110611110111231103001010120100001011?1

*G_cristatus* 0001020020012211000100110000000011?1?1000100000000000001011000?101000?10??1?00001?05001130?102010{01}0010????1011011111?1

*G_rhamphastinus*  000102002001221100011011000000001101?1000100000000000001010000?101000110??1000011?05001200??020000001012?11011??1011?1

*Dsungaripterus*  000102002001221??0?1001?20030000110101000100000000000001011000110110111010110000?00500???0???20?0?00??123?1011011????1

Tatal pterosaur 000102002001221??0?1001?200300001101?10001000000000000010110001101100?????11?000200500?130?1120?0100?0????10110110????

*Noripterus*

?????????0???????????????????????????1?0?1????000???000??1100?11010????????????0?00500?030?1120?0100?01????0110?1111?1

*Lonchodectes*  000?????{01}0????1?????????????????????1?0011000{01}0000000000000000101112???????1?0?0?1050??????????????????????0011???????

*Tapejara*  100200004011221??0?1101?011300000102010001000100000000100000001011120???10110001?00500?1?0??120?1?00?0102??0000?10?1??

*Tupandactylus* 100200104011221??0?1101?0114000001?2?1000100010000000010000000????????????????????????????????????????????????????????

*Sinopterus*  1002000000?1221??0?1101?011300000101?10001000100000000100000001?111201101011??011??5?0113021?20011002010211001?11011?1

*Huaxiapterus* 1002000040?1221??0?1100?01140000010101000100010000000010000000??1112?110??110??111050011302112001100{12}0???11001??10???1

*Shenzhoupterus*  000202110011221??0?1101?0115000001?2?1000100100000000010000000??11120?10??11?0011??5001230?12200110020????100????0?1?1

*Chaoyangopterus* 0002?2?100??221????1?????????????1????00010010?000000010000000??1112??1???1????11??????13?2122?0100020????100???10?1?1

*Tupuxuara*  000201103011221??0?1101?011500000102110001000000000000100000001101101110101100011105001030212200110020102?1001111011??

*Thalassodromeus*  000201103011221??0?1101?01150000010211000100000000000010000000????????????????????????????????????????????????????????

*Quetzalcoatlus* 00021310501?221??0?1001?011??????102010001001000000000100000001021121????0110001110500?1302?2300111020?????0011120?1??

*Zhejiangopterus* 000202100011221??0?1100?0110000101?2?1000100100000000010000000??21121110?011?0011?05?0123?21?3001110?010201001?12011?1

*Azhdarcho* 000??????0????1???????????????????????000100???0000000100000001021121??????????1?1050????0????????10???????00111??????

*Zhenyuanopterus* 0002121050112210000101101010000001?0?100010000100000010011000011110011101?11?1112??10110111111110200101???110????01101

*Changchengopterus*

????????????????????????????????????????????????????????????????00??0001??110?0110?0?0?1202100??0100????????0????11011

*Moganopterus*  000112103010221000010100100400?0?1?0?1000100001000000100000100102112??????????????????????????????????????????????????

*Feilongus* 000112101010221000010100100011???1?21?000100001000000100000100102112??????????????????????????????????????????????????

*Fenghuangopterus* 00010000000??1?????0????0??????0?1?0?100?11000?00011?100000100?000??000120210000?002?00030?101????000?????0??????????0

*Gladocephaloideus* 00011210001002??0011010110111101?2?2?1001100000000000100000100?011120???3010000?1?1110?03011010101?000?????01?10?11101

*Guidraco*  01011200000012110001011110010001?102?1001100001000000100100100110????????????????????????????????????????????????????1

*Beipiaopterus*

????????????????????????????????????????????????????????????????201?1110??1?0?0?2??11010301??101021020????0?0??0111101

*Elanodactylus*

??????????????????????????????????????????????????????????????1?2112{01}???4110000??01110???02101?20?00?11122????????1101

*Pterofiltrus* 000112100000221100110101100001????02??00110000?000000100000100???????????????????????????????????????????????????????1

*Cathayopterus*  00011220000????1??0100???????????????00011000??000000100000100???????????????????????????????????????????????????????1

References

S1 Lü JC, Unwin DM, Jin X, Liu Y, Ji Q. Evidence for modular evolution in a long-tailed pterosaur with a pterodactyloid skull. Proceedings of the Royal Society B 2010; 227:383–389.

S2. Vila Nova BC, Sayão JM, Neumann V H M L, Kellner AWA. Redescription of Cearadactylus atrox (Pterosauria, Pterodactyloidea) from the Early Cretaceous Romualdo Formation (Santana Group) of the Araripe Basin, Brazil. Journal of Vertebrate Paleontology 2014; 34: 126-134.
